# Effects of non-sinusoidal pitching motion on the propulsion performance of an oscillating foil

**DOI:** 10.1371/journal.pone.0218832

**Published:** 2019-07-01

**Authors:** Zhanfeng Qi, Jingsheng Zhai, Guofu Li, Jiazhong Peng

**Affiliations:** 1 School of Marine Science and Technology, Tianjin University, Tianjin, China; 2 National Ocean Technology Center, Tianjin, China; University of New South Wales, AUSTRALIA

## Abstract

Numerical simulations have been used in this paper to study the propulsion device of a wave glider based on an oscillating hydrofoil, in which the profile of the pitching and heaving motion have been prescribed for the sake of simplicity. A grid model for a two-dimensional NACA0012 hydrofoil was built by using the dynamic and moving mesh technology of the Computational Fluid Dynamics (CFD) software FLUENT and the corresponding mathematical model has also been established. First, for the sinusoidal pitching, the effects of the pitching amplitude and the reduced frequency were investigated. As the reduced frequency increased, both the mean output power coefficient and the optimal pitching amplitude increased. Then non-sinusoidal pitching was studied, with a gradual change from a sinusoid to a square wave as the value of β was increased from 1. It was found that when the pitching amplitude was small, the trapezoidal pitching profile could indeed improve the mean output power coefficient of the flapping foil. However, when the pitching amplitude was larger than the optimal value, the non-sinusoidal pitching motion negatively contributed to the propulsion performance. Finally, the overall results suggested that a trapezoidal-like pitching profile was effective for the oscillating foil of a wave glider when the pitching amplitude was less than the optimal value.

## Introduction

In recent years, the use of renewable energy originating from the ocean, such as wave, sunlight and tidal, has been extensively explored, and more and more researchers have focused on using the energy of the ocean to drive marine vehicles for oceanic research and monitoring. The first wave glider, developed by Liquid Robotics Corporation, relies on the ocean’s energy. The innovation of the wave glider is its ability to harvest the abundant energy in ocean waves to provide essentially limitless propulsion [1]. Therefore, it is necessary to research on the system’s hydrodynamic characteristics in order to optimize the propulsion performance of the wave glider.

**Fig 1:**
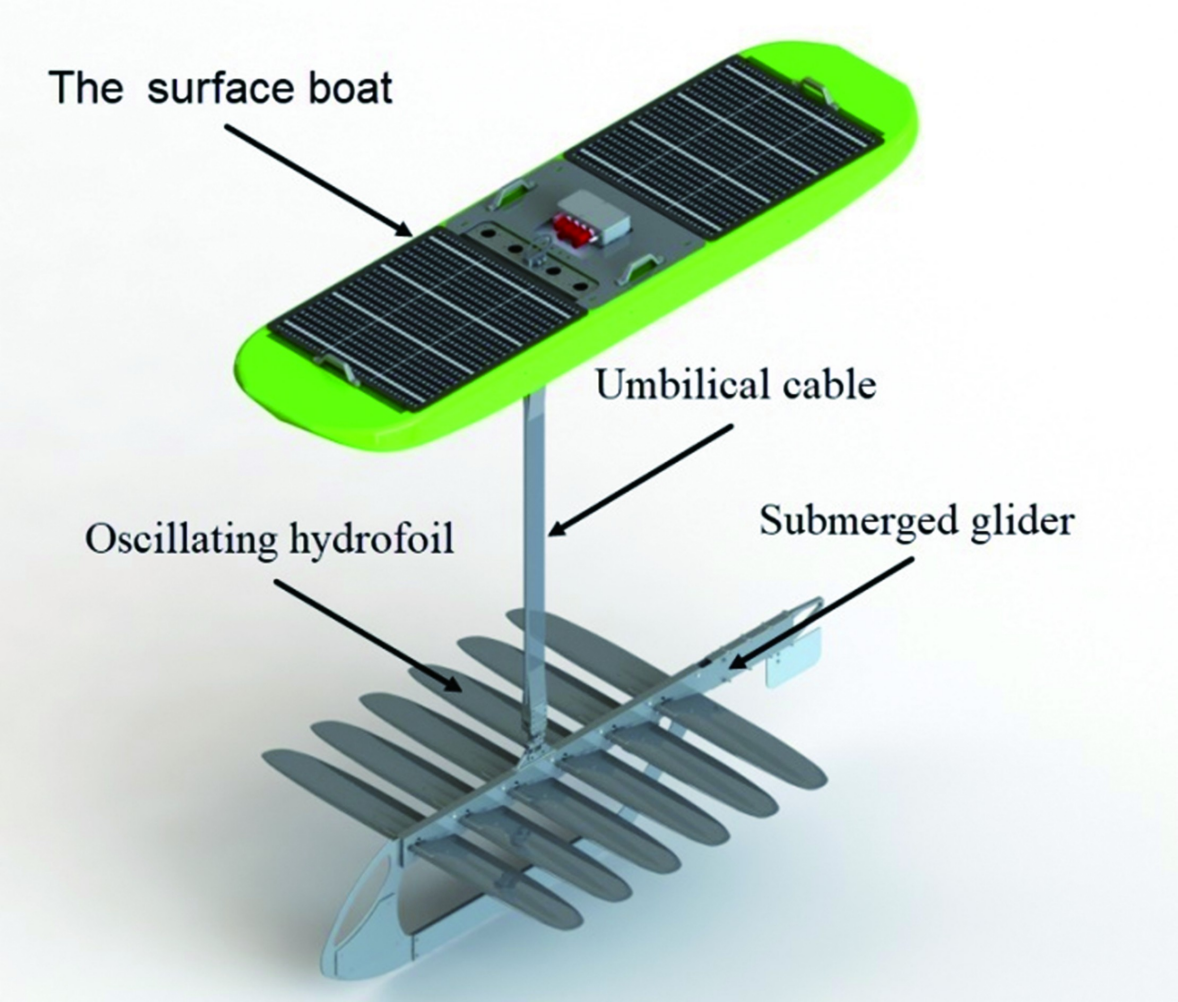
The wave glider platform developed by National Ocean Technology Center.

**Fig 2:**
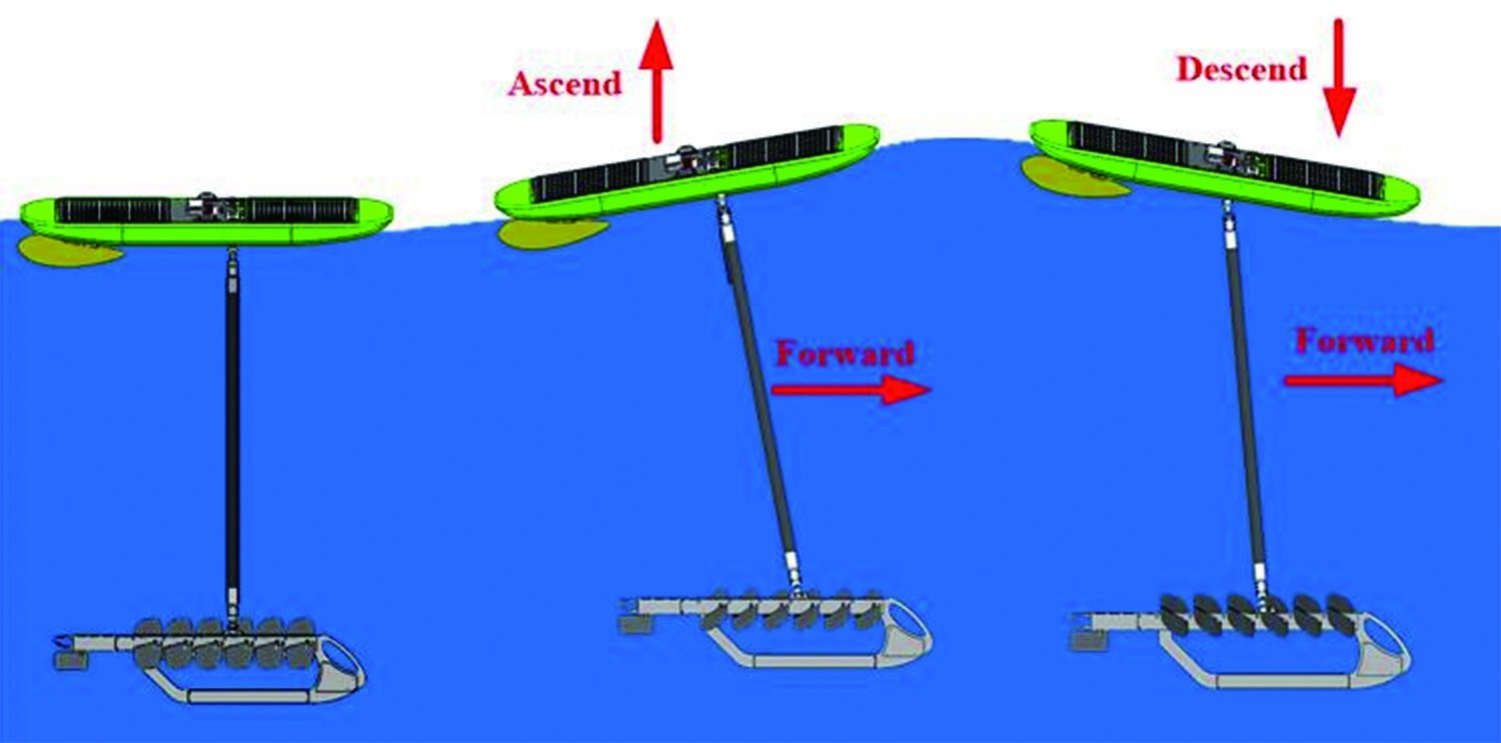
The operating principles of the wave glider.

The wave glider used in this paper was developed by National Ocean Technology Center, originally manufactured by Liquid Robotics Corporation, is a new kind of wave-propelled, persistent unmanned surface vessel, it is comprised of a surface boat attached to a glider via an umbilical cable, Fig 1. The wave glider is propelled by a unique, purely mechanical, propulsion system. When a wave passes by the surface boat, this causes the hull to move up and down which causes vertical movement of the submerged glider. The submerged glider’s foils interact with the comparatively still waters, and converts a portion of the vertical motion into forward thrust; the principles of its operation have been shown in Fig 2. Under the continuous effect of the waves, the submerged glider drags the surface boat and moves forward continuously. This hybrid drive principle is the crucial aspect of the wave glider’s design.

The propulsion mechanism of the wave glider uses an oscillating hydrofoil to convert the heaving motion into propulsion under the action of waves, and it is a passive propulsion device. Nowadays, the oscillating hydrofoil has been extensively studied for use as propulsion. Based on the potential flow approach, Wu [2–5] studied the waving motion of a two-dimensional flexible plate, and then gave the optimal oscillation motion and shape parameters in his research. Kubota and Kato [6, 7] developed both linear and nonlinear theories to estimate the propulsive performance of flexible and rigid oscillating propulsion. Triantafyllou et al. [8] found that oscillating foils produced thrust through the development of a jet-like average flow, and only at the frequency of maximum amplification can the optimal efficiency be obtained in which a stable co-existence of the jet profile and the large-scale patterns is ensured, the frequency is in the range of 0.25 to 0.35; the experiments confirmed this result.

At present, most researchers have studied active propulsion devices or bionic propulsion devices [9–12]. Licht and Triantafyllou [9] developed a biomimetic autonomous underwater vehicle with four flapping hydrofoils. Low et al. [10] designed a dual-purpose robotic turtle to imitate locomotion behaviors of Cheloniidae. Xu et al. [11] researched on the bionic turtle forelimb’s hydrofoil propulsion principle. Seo et al. [12] presented an autonomous underwater vehicle by constructing coupled non-linear oscillators. Anderson et al. [13] studied thrust-producing harmonically oscillating foils through force and power measurements, and classified the principal characteristics of the flow in and around the wake of the foil. Read et al. [14] and Hover and Triantafyllou [6, 15] noticed that propulsive performance of a harmonically heaving and pitching foil was degraded by a breakdown of the reverse von karman street in the angle of attack profile, the corresponding thrust coefficient of cosine profile is nearly four times larger than that of harmonic heave motion. Young and Lai [7] analyzed the effects of the oscillation frequency and amplitude on the wake of a plunging airfoil by using a compressible two-dimensional Navier-Stokes solver. The above literatures indicated that the Strouhal number, heave amplitude and maximum angle of attack were the main factors affecting the propulsive performance of a sinusoidal oscillating hydrofoil for active propulsion devices, these factors affect the trailing vortex, propulsion efficiency and average thrust coefficient of the oscillating hydrofoil.

Due to its complex mechanics and poor reliability, the active oscillating hydrofoil has not been considered as a practical replacement for the traditional thruster [16]. A passive system like the wave glider’s propulsion device has a simpler mechanical structure and is more practical than active propulsive devices. A steady-state simulation using CFD has been used to analyze the lift and drag characteristics of wave glider's hydrofoil [17–18]. Jia et al. [17] analysed the effects of hydrofoil profile, maximum rotating angle and interval space of the multiple flapping hydrofoils on propulsion performance, and noted that the propulsion performance of NACA0006 hydrofoil is better than that of flat hydrofoil. Zheng et al. [18] found that the horizontal component force is maximum when attack angle reaches 45 degrees. A numerical method using CFD and Maxsurf software has been applied to predict calm water resistance for the surface boat of wave glider [19]. After these studies, Kraus and Bingham [20] established a simplified dynamic model appropriate to understand the capabilities and limitations of the wave glider's novel propulsion mechanism; they noted that the wave glider can be used for applications requiring precise estimation. Tian et al. [21] applied the D-H approach and Lagrangian mechanics to simulate the performance of the wave glider’s motion. Yang et al. [22] presented an unsteady Reynolds averaged Navier-Stokes numerical simulation to predict the wave glider’s dynamic performance in head seas. They noted that the surge force acting on the surface boat and the passive eccentric rotation law of the hydrofoils were the main factors affecting the propulsion efficiency of the wave glider. Liu et al. [23] used a CFD method based on Navier-Stokes equations to analyze the hydrodynamic performance of 2D tandem asynchronous flapping foils with different parameters of non-dimensional wave condition. The above literatures have studied the propulsion performance and influencing factors of the oscillating hydrofoil of the wave glider, the key contributions of these researches have been given in Table 1. However, there is still a lack of numerical simulation of the effect of non-sinusoidal pitching profile on the propulsion performance of the oscillating hydrofoil.

**Table 1:** Highlights of earlier studies on wave glider.

Reference	Year	Model/Shape	Merits
**Jia et al.**	2014	2-D numerical, NACA0006/Plate	First simulation of the interval space of flapping foils
**Zheng et al.**	2015	2-D numerical, NACA0006	Point out the optimal angle of attack for thrust
**Tian et al.**	2015	3-D numerical,	First simulation the motion based on D-H approach
**Yang et al.**	2018	3-D numerical, NACA0012	(a)First analyze the spring effect (b)Simulation of the surge force acting on the surface boat
**Liu et al.**	2016	2-D Numerical, NACA0012	First simulation under non-dimensional wave condition

Recently, researchers have focused on trapezoidal-like pitching motion hydrofoils to improve the extraction performance of marine current turbines. Xiao et al. [24], Jian et al. [25] and Teng et al. [26] have addressed that modified pitching motion that can improve the efficiency of energy extraction in some cases. Wang et al. [27] have studied the effects of non-sinusoidal pitching motion on the hydrodynamic load and power input performance by a full active pitching foil. Effects of pitching motion profile on energy harvesting performance of a semi-active flapping foil are numerically studied using immersed boundary method by Li et al. [28]. They found that increasing the value of β was effective to enhance energy harvesting efficiency for cosinusoidal pitching motion. Qadri et al. [29] have investigated the energy extraction performance through flapping foil which is driven by incoming free-stream flow. Free-stream velocity, pitching amplitude and inertial mass block load effects on the energy harvesting performance of passively actuated flapping energy harvester were discussed. They noted that the energy harvester is sensitive to changes in inertial loads. According to the mechanism of the wave glider, the hydrofoil of the wave glider can be defined as an oscillating hydrofoil. The aim of this research was to improve the wave glider’s propulsion performance by modifying the pitching profile of the oscillating hydrofoil.

Firstly, by comparing with the motion model of a traditional oscillating hydrofoil, the heaving and pitching motion models of the oscillation foil have been given in this paper. Then, the dimensionless parameters of propulsion performance have been defined, and the independence of the grid and time discretization has been verified. Afterwards, the effects of pitching amplitude and reduced frequency on sinusoidal pitching motion have been studied, and the effects of non-sinusoidal pitching motion on the propulsion performance and vortex structure of oscillating hydrofoil have been further analyzed. Finally, the effects of the pitching amplitude and the non-sinusoidal pitching profile on the propulsion performance of the flapping foil have been systematically discussed.

## Motion model and kinematics

### Kinematic motion of the hydrofoil

**Fig 3:**
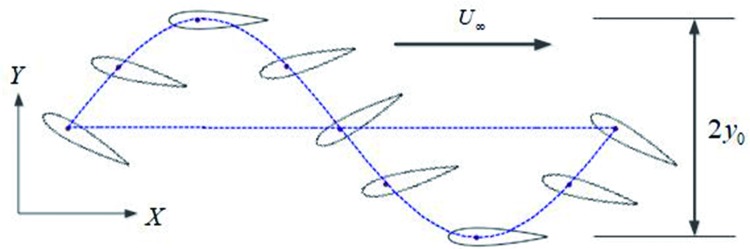
The sinusoidal pitching profile of a traditional oscillating hydrofoil.

Several underwater vehicles that use flapping foils have been built by the author [30]. The traditional motion model is described as a foil undergoing harmonic flapping which is a combination of a heave translation and a pitch rotation [6], as shown in Fig 3. The hydrofoil’s motion is limited to simple harmonic flapping defined by:

$$\left\{ \begin{array}{l} y(t)=y_{0}\sin(2\pi ft)\\ \theta (t)=\theta_{0}\sin(2\pi ft+\Phi ) \end{array}\right.$$ (1)

Where *y*_0_ is the heaving amplitude, *θ*_0_ is the pitching amplitude, d is the thickness of the hydrofoil, and c is the foil’s chord length. The pitching axis is located at 1/3 of the chord length from the leading edge, *f* is the heaving frequency, f* is the reduced frequency, f* = *fc*/*U*_∞_. Φ is the phase between the heave and pitch motions and is kept constant at π/2, and *U*_∞_ is the free stream velocity far upstream of the oscillating hydrofoil.

**Fig 4:**
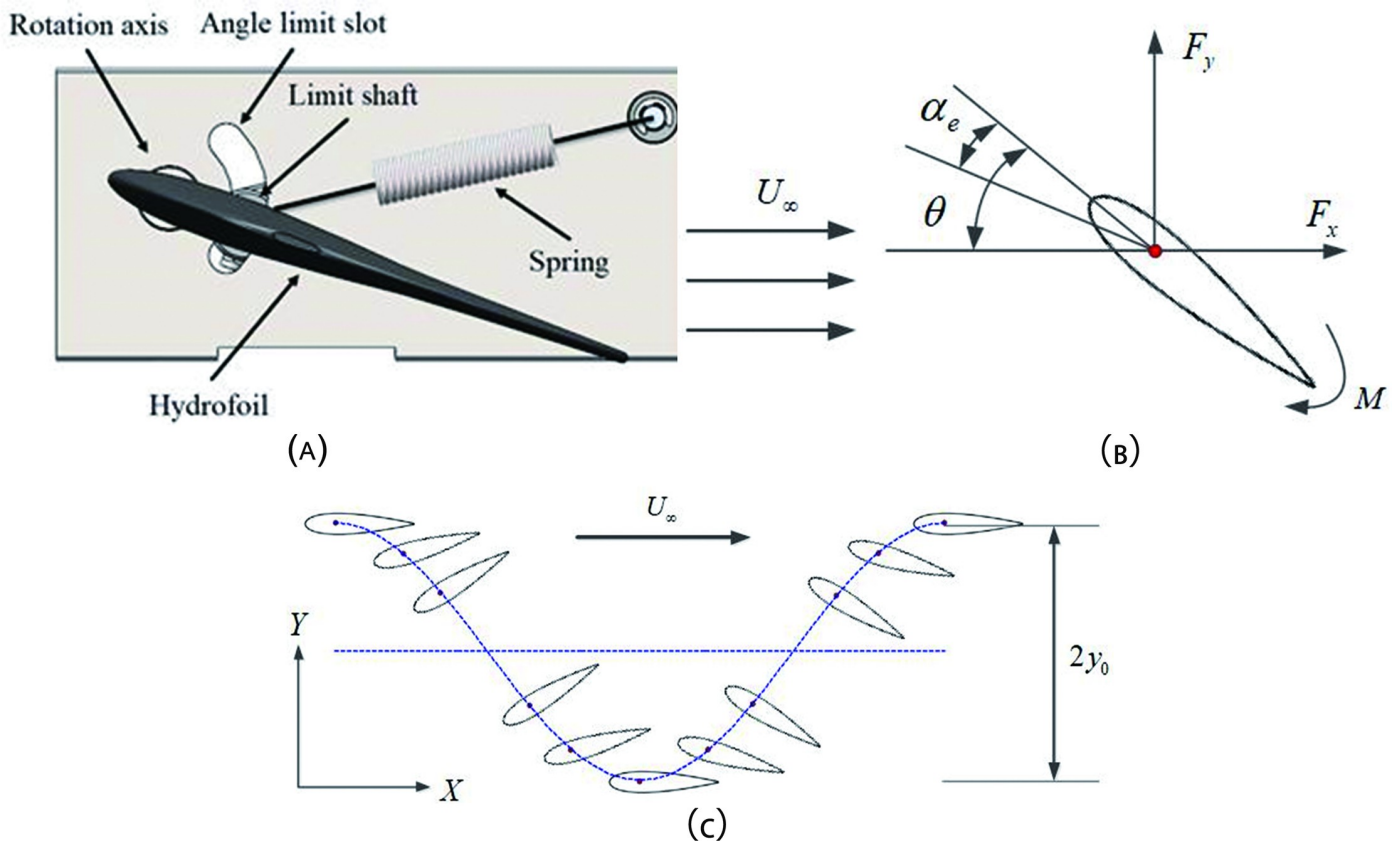
The motion model of the wave glider, (a) Passive oscillating foil (b) Schematic of angles and forces on an oscillating foil (c) non-sinusoidal pitching profile.

Compared with Figs 3 and 4, it is obvious that the passive oscillating hydrofoil has a simpler structure than the active device. According to the basic mechanics of locomotion for the wave glider, this study has proposed a non-sinusoidal pitching motion of the oscillating hydrofoil, as shown in Fig 4. For the sake of simplicity, the prescribed motion mode, whereby the foil experiences a non-sinusoidal pitching motion and sinusoidal heaving motion simultaneously has been used for the analysis. The hydrofoil’s heaving motion can be expressed as:

$$y(t)=y_{0}\sin(2\pi ft+\Phi )$$ (2)

$$V_{y}(t)=y_{0}2\pi f\cos(2\pi ft+\Phi )$$ (3)

Where *V*_*y*_ is the heaving velocity. The non-sinusoidal pitching profile of the oscillating hydrofoil can be defined as:

$$\theta (t)=\{\begin{array}{l} \theta_{0}\sin(2\pi f\beta t)\;t\in (0,\frac{1}{4\beta f})\\ \theta_{0}\;t\in (\frac{1}{4\beta f},\frac{1}{4f}(2‑\frac{1}{\beta }))\\ \theta_{0}\sin(\pi /2+2\pi f\beta (t-(T/2-T_{\beta }/4)))\;t\in (\frac{1}{4f}(2‑\frac{1}{\beta }),\frac{1}{4f}(2+\frac{1}{\beta }))\\ -\theta_{0}\;t\in (\frac{1}{4f}(2+\frac{1}{\beta }),\frac{1}{4f}(4-\frac{1}{\beta }))\\ \theta_{0}\sin(3\pi /2+2\pi f\beta (t-(T-T_{\beta }/4)))\;t\in (\frac{1}{4f}(4-\frac{1}{\beta }),\frac{1}{f}) \end{array}$$ (4)

**Fig 5:**
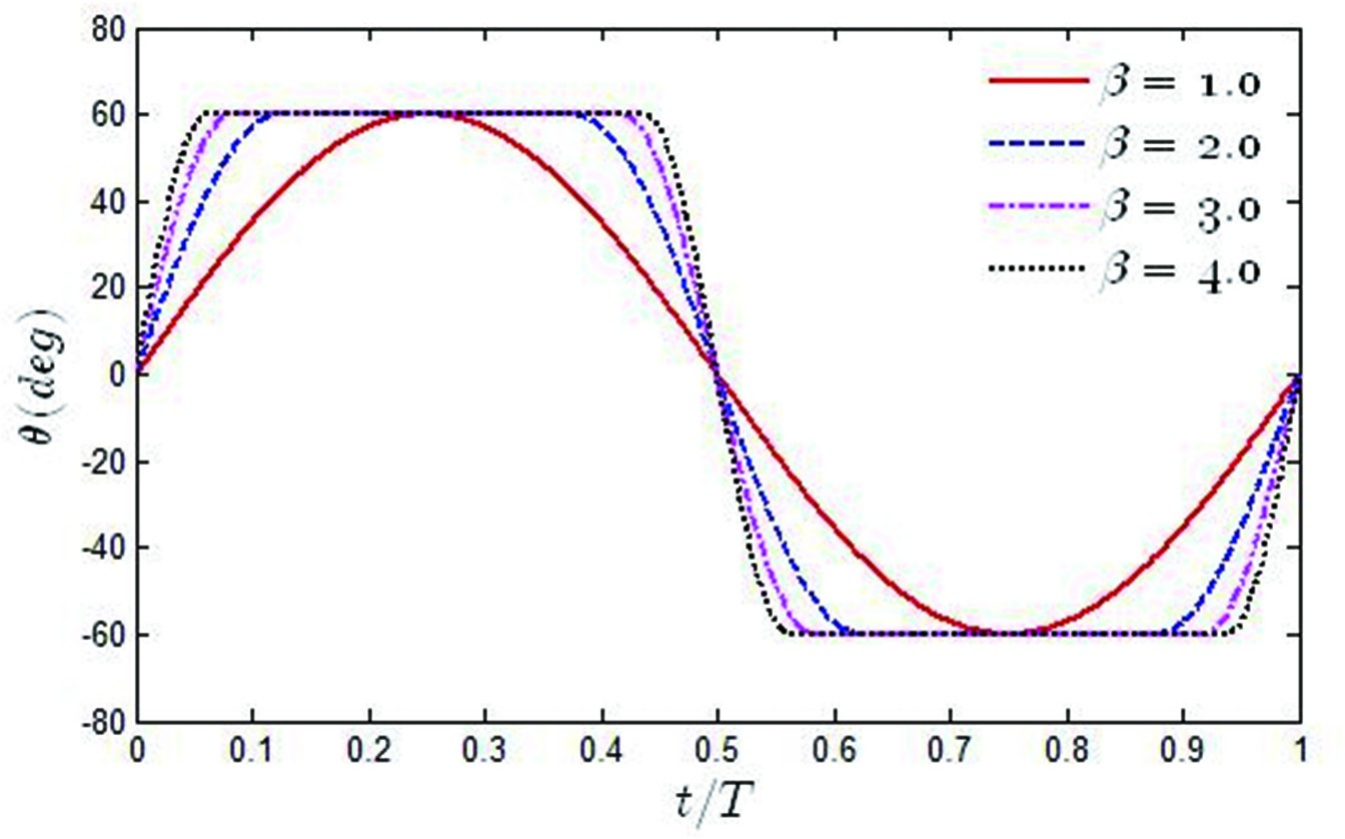
Time variation of *θ*(*t*) for different values of β in one period.

Where *f*_*p*_ is the pitching frequency, *β* is the ratio of the pitching frequency to the heaving frequency, β = *f*_*p*_/*f*. It is should be noted that when β→∞, the pitching profile of the oscillating hydrofoil tends from sinusoidal to trapezoidal The time variation of the pitching angle *θ*(*t*) for some typical values of β has been shown in Fig 5. With the increase of β, the pitch angle *θ*(*t*) tends to a square wave.

Based on the motion imposed and the upstream flow conditions, as shown in Fig 4B, the effective angle of attack α_*e*_(*t*) and the heaving induced angle α_*h*_(*t*) can be defined as follows [31–32]:

$$\alpha_{e}(t)=\theta (t)-\alpha_{h}(t)$$ (5)

$$\alpha_{h}(t)=\arctan(V_{y}/U_{\infty })$$ (6)

### Parameterization of the propulsion performance

The dimensionless coefficients for thrust, lift and moment are defined as:

$$C_{X}(t)=F_{X}(t)/\frac{1}{2}\rho U_{\infty }^{2}c$$ (7)

$$C_{Y}(t)=F_{Y}(t)/\frac{1}{2}\rho U_{\infty }^{2}c$$ (8)

$$C_{M}(t)=M(t)/\frac{1}{2}\rho U_{\infty }^{2}c^{2}$$ (9)

Where *ρ* is the fluid density, *F*_*X*_ is the horizontal hydrodynamic force, *F*_*Y*_ is the vertical hydrodynamic force and *M* is the torque at the pitching axis, which is shown in Fig 4B.

The instantaneous input power *P*_*I*_(*t*) can be defined as:

$$P_{I}(t)=P_{y}(t)+P_{\theta }(t)=F_{Y}(t)V_{y}(t)+M(t)\Omega (t)$$ (10)

Where P_y_ is the heaving contribution to the input power, P_θ_ is the pitching contribution to the input power and Ω is the pitching velocity. The input power coefficient C_PI_(t) and the output power coefficient C_PO_(t) can be defined as:

$$C_{PI}(t)=P_{I}(T)/\frac{1}{2}\rho U_{\infty }^{3}c$$ (11)

$$C_{PO}(t)=F_{X}(t)U_{\infty }/\frac{1}{2}\rho U_{\infty }^{3}c$$ (12)

The propulsion efficiency *η* [16, 32] is defined as the ratio of the output power coefficient over the input power coefficient which can be written as:

$$\eta =\frac{\bar{C_{PO}}}{\bar{C_{PI}}}$$ (13)

Where the over line denotes averaging over one period of oscillation. If T is the period of oscillation, $\bar{X}$ is the time-average value of any time varying function *X*(*t*):

$$\bar{X}=\frac{1}{T}\overset{T}{\underset{0}{\int }}X(t)dt$$ (14)

## Numerical modeling and validation

The Finite Volume Method (FVM) program Fluent was used to solve the unsteady-Reynolds-averaged-Navier-Stokes (URANS) equations for the hydrofoils [31–33]. The simulations were performed with a high Reynolds number (*Re* = 42000) in order to ensure that the boundary layers are turbulent, which allows us to solve the simulation with a turbulence model in fully turbulent mode. The one-equation Spalart-Allmaras turbulence model was chosen for URANS simulation of oscillating foil [31–32, 34–36]. The SIMPLE algorithm was selected to be used for the pressure-velocity coupling. Second order schemes were used for pressure, momentum and turbulent viscosity resolution. The second order implicit scheme was used for the unsteady formulation. Absolute convergence criteria of 1e^-5^ were set for the *x*-velocity, *y*-velocity, continuity and nut. The inner grid motion and the inlet condition were controlled in Fluent using user-defined functions (UDFs). The user-defined functions (UDFs) have been given in S1 File.

**Fig 6:**
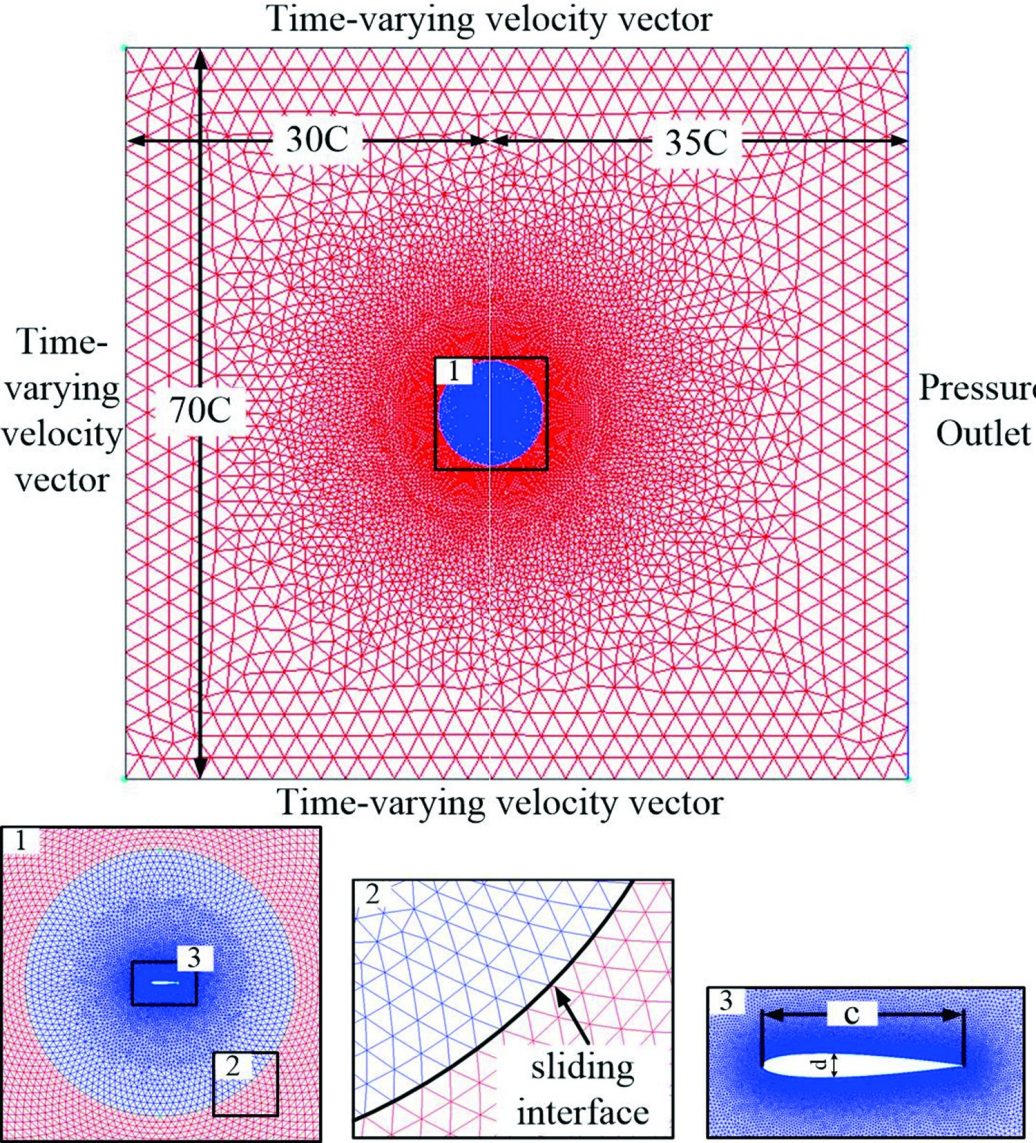
Mesh details of the oscillating foil.

The mesh strategy for the oscillating hydrofoil’s computations has been summarized in Fig 6. The interface was located at 5*c* around the hydrofoil. The far field boundary was located at 35*c* and 40*c* from the hydrofoil. The sliding interface was used to allow relative motion between the inner grid and the outer grid, the inner grid around the hydrofoil was pitching in the rigid body, the outer grid outside the interface was not moving. This strategy required the time-varying velocity inlet condition to be specified on the upstream, top and bottom boundaries, and the outlet condition was set to uniform static pressure.

**Fig 7:**
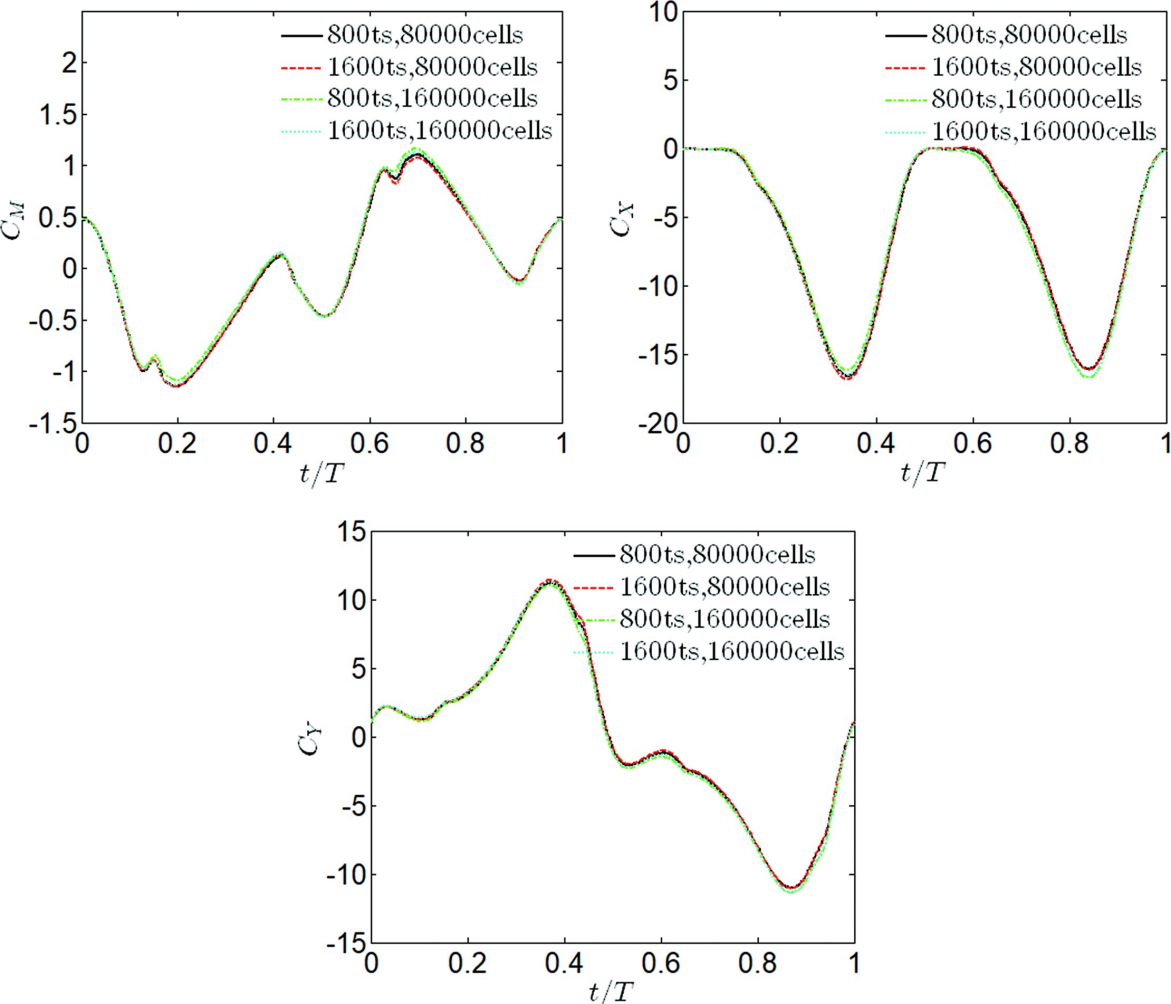
Evolution of *C*_*X*_, *C*_*Y*_ *and C*_*M*_ for the different levels of cells and time steps at *θ*_0_ = 57° and f* = 0.34.

In order to verify the meshing strategy, two different meshes were simulated (80000 cells and 160000 cells), as well as two different time steps (800ts/cycle and 1600ts/cycle). The simulations for each cycle require about 5 hours to complete on a i7-7820 processor. According to different parameters of reduced frequency, it typically requires 6–12 oscillation periods to get a periodic solution from the initial state. The criterion of the periodic solution is that the variations are less than 1% on cycle averaged values. The comparison of time histories of force coefficients of the last two flapping cycles (160000 cells and 1600ts/cycle) has been given in S2 File. The time histories for *C*_*X*_, *C*_*Y*_, and *C*_*M*_ have been shown in Fig 7, and the numerical results of $\bar{C_{PO}},\;\bar{C_{PI}}$ and *η* have been shown in Table 2. Through comparison of the above results and taking into consideration the efficiency of the simulation, the medium mesh and time resolution were chosen for the study.

**Table 2:** The numerical results for the different levels of cells and time steps (*θ*_0_ = 57, f* = 0.34).

Cell number	Time step	$\bar{C_{PO}}$	$\bar{C_{PI}}$	*η*
**80000**	800	6.264	21.573	29.038
**80000**	1600	6.293	21.752	28.930
**160000**	800	6.327	21.893	28.9
**160000**	1600	6.407	22.210	28.846

**Fig 8:**
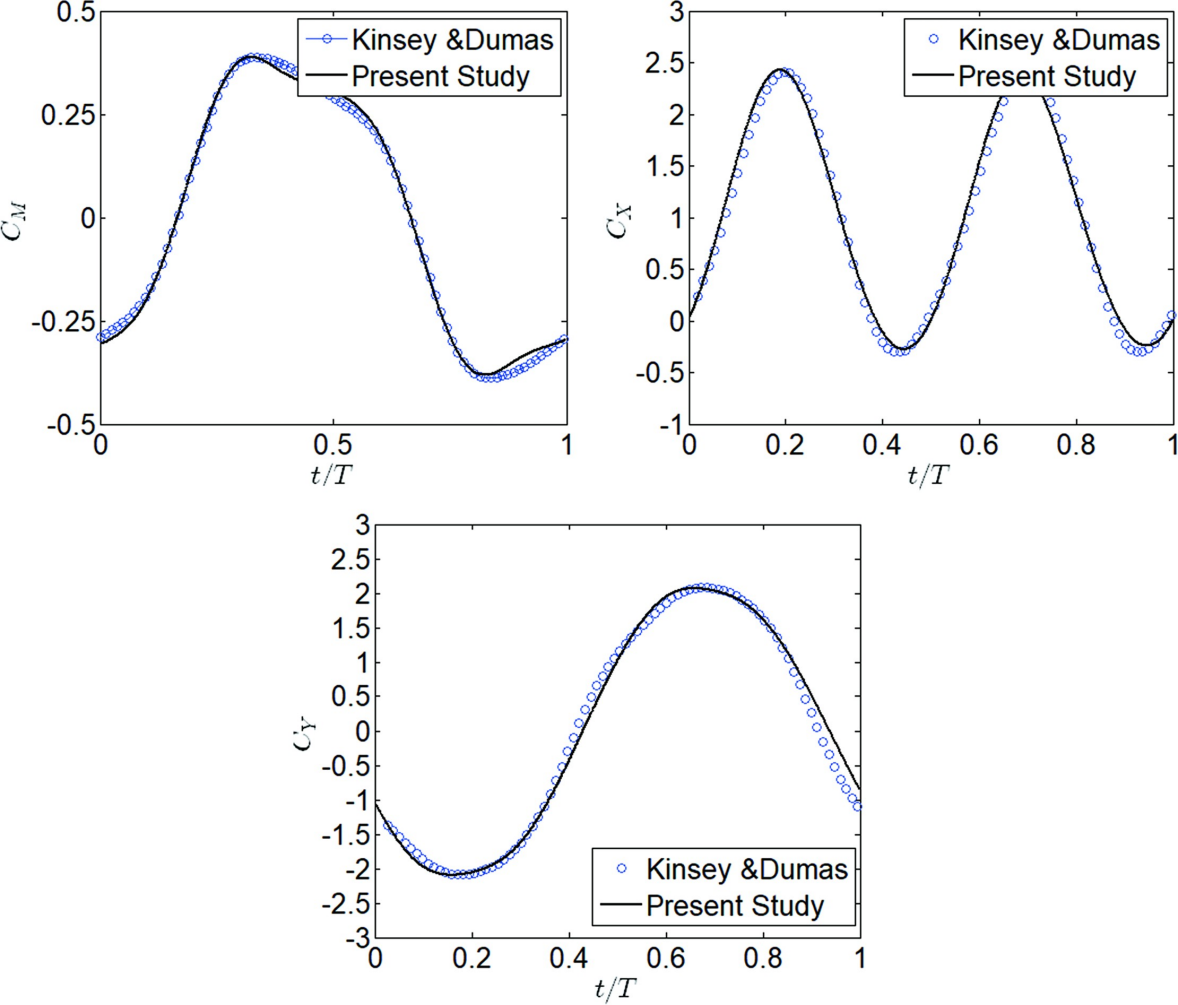
Comparison of *C*_*X*_, *C*_*Y*_ and *C*_*M*_ from the literature [30] and this study.

The performance of oscillating hydrofoils extracting energy from water currents has been investigated by Kinsey and Dumas using URANS numerical simulations [31, 35, 37–38].The mesh strategy and simulation parameters settings were verified via comparison with the experimental data. The simulation conditions were for a NACA0015 cross-section shape, with a chord length c = 0.24m, a heaving amplitude of *y*_0_ = *c*, a pitching amplitude of *θ*_0_ = 60° and a reduced frequency of f* = 0.14 [38]. In this study, the simulation was carried out according to the parameters from the literature. The time histories of *C*_*X*_, *C*_*Y*_, *and C*_*M*_ have been compared with the literature and shown in Fig 8. It was found that the results in the present study were consistent with that of the literature [38], which demonstrated the accuracy of the simulation strategy.

## Results and discussion for the oscillating hydrofoil

In this section, the numerical results of the sinusoidal and non-sinusoidal pitching motions of the oscillating hydrofoil have been described. Firstly, the effects on the propulsion performance in sinusoidal pitching motion were analyzed. Then the impact of the trapezoidal-like pitching profile on the oscillating hydrofoil’s propulsion performance was studied, focusing on the analysis of both small and large pitching amplitudes. Referring to the parameters of the wave glider and typical sea conditions [39–40], the main parameters of which have been shown in Table 3.

**Table 3:** The main parameters in this study [39–40].

Parameters	Scale
Chord length, *c*	0.17m
Hydrofoil thickness, *d*	0.02m
Heaving amplitude, *y*_0_	3*c*
Reduced frequency, f^*^	0.068–0.612
Hydrofoil shape	NACA0012
Free stream velocity, *U*_∞_	0.25m/s
Pitching amplitude, *θ*_0_	20–70°
Ratio of pitch frequency to heave frequency, *β*	1–4

### Sinusoidal pitching motion

**Fig 9:**
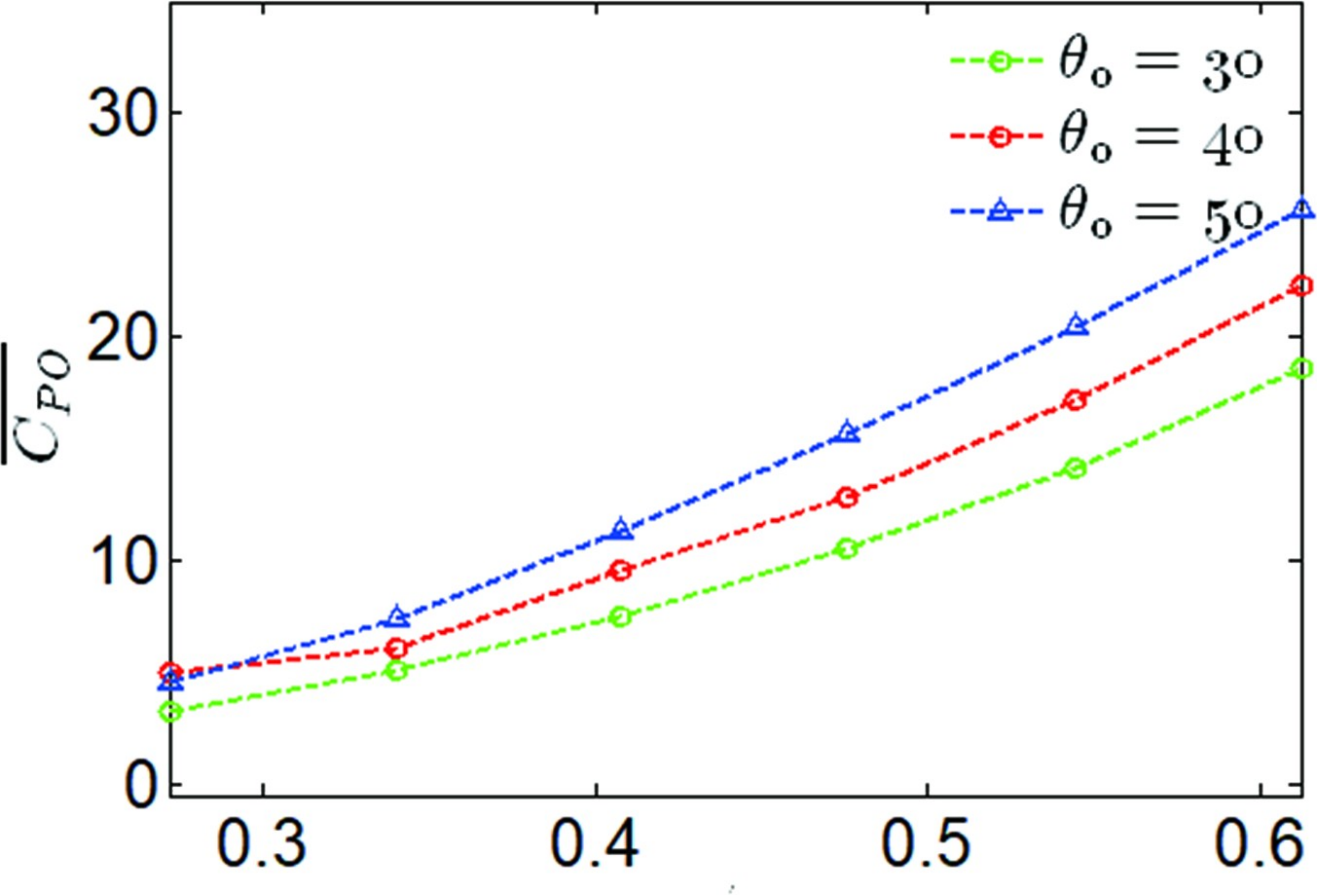
Variations of the mean output power coefficient with reduced frequency for different pitching amplitudes.

The relationship between the mean output power coefficient and the heaving frequency for ***θ***_**0**_ = 30,40,50° and f* = 0.272−0.621 has been shown in Fig 9. This has shown that as the reduced frequency increased, the mean output power coefficient increased linearly, which means the propulsion performance was constantly improving. After f* = 0.34, the mean output power coefficient growth rate for ***θ***_**0**_ = 40° decreased, compared to ***θ***_**0**_ = 50°. According to Eqs (5) and (6), the reduce frequency affected the heaving induced angle and the effective angle of attack directly, which results in this phenomenon.

**Fig 10:**
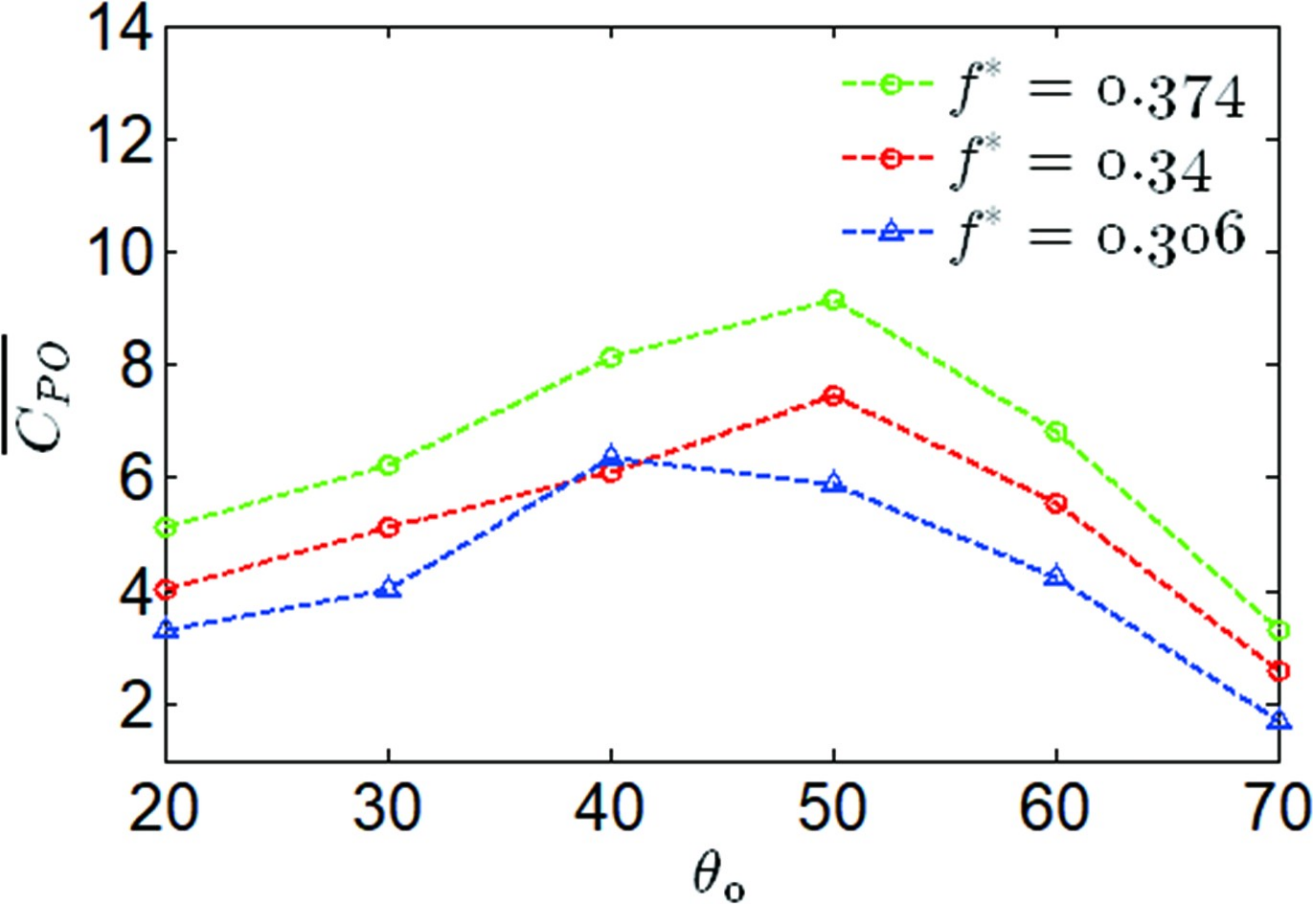
Variations of the mean output power coefficient with pitching amplitude for different reduced frequencies.

The variations of the propulsion performance versus the pitching amplitude at ***θ***_**0**_ = 20−70° and f* = 0.306,0.34,0.374 have been shown in Fig 10. It was noted that the maximum mean output power coefficient had a significant relationship with the values of ***θ***_**0**_ and f*. When f* increased from 0.306 to 0.374, the highest mean output power coefficient increased by 16.8% and 44%, and the optimal pitching amplitude changed from 40° to 50°. It was also noted that there was a linear increase in the mean output power coefficient at ***θ***_**0**_<40°, and the highest mean thrust force coefficient was achieved at ***θ***_**0**_ = 40° for f* = 0.306, a similar phenomenon was also obtained at a reduced frequency of 0.34 and 0.374. This predicted that the response to the variation of pitching amplitudes for the mean output power coefficient of f* = 0.374 would be larger than that of f* = 0.306,0.34, as shown in Fig 10.

The above analysis has shown that the propulsion performance of the oscillating hydrofoil is very sensitive to the heaving frequency, and the effect of the pitching amplitude on the propulsion performance is also related to the heaving frequency.

### Non-sinusoidal pitching motion

According to the previous studies, the effects of pitching amplitude and the reduced frequency on the propulsion performance of the oscillating hydrofoil has been fully studied, however there have been a few papers that focused on the effects of pitching profile on the propulsion performance. Xiao and Liao [41] have investigated the effect of effective angle of attack profile on the propulsion performance of a NACA0012 oscillation hydrofoil. The pitching and heaving motions were modified from the conventional harmonic sinusoids. They found that the imposed modification on pitching motion can induce the improved the thrust performance. Hover et al. [15] compared directly the thrust performance obtained with four specific angle of attack profiles, and noted that the cosine angle of attack profile achieves a very clear improvement in efficiency. In this paper, the non-sinusoidal pitching profile was designed referring to wave glider swinging hydrofoil propulsion principle and the pitching limit angle was considered in pitching motion.

In the case of the heave frequency and fixed heave amplitude, the relationship between pitching amplitude, pitching profile and propulsion performance has been analyzed. The optimal pitching amplitude was found, as shown in Fig 10, which reached the highest value of the mean output power coefficient. With the purpose of understanding the effects of the trapezoidal-like pitching profile on the propulsion performance of the oscillating hydrofoil, two cases were analyzed by choosing small and large amplitudes, and their different responses to the propulsion performance were found.

#### Small pitching amplitude

**Fig 11:**
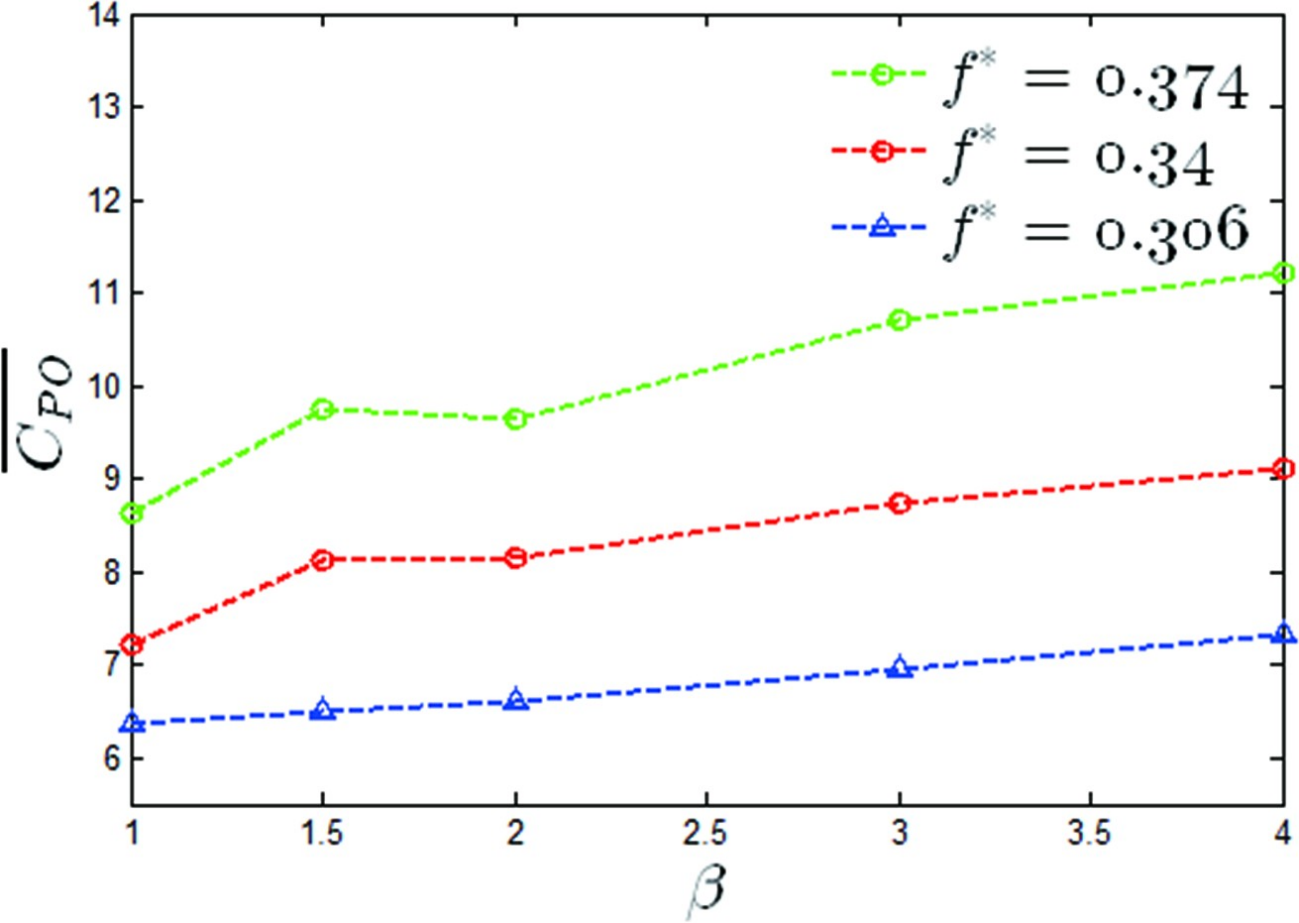
Variations of the mean output power coefficient with *β* (*θ*_0_ = 40°, f* = 0.306,0.34,0.374).

**Fig 12:**
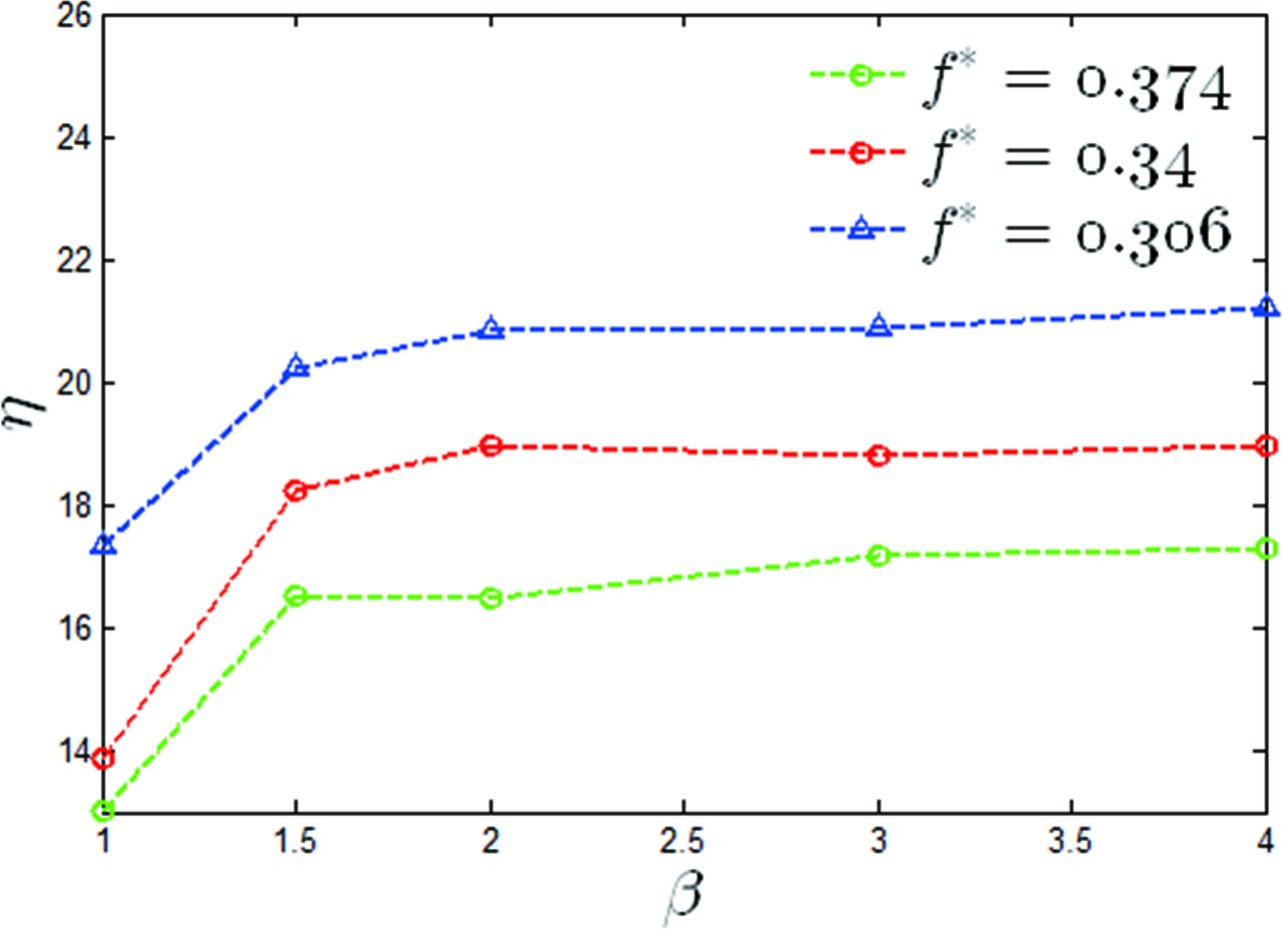
Variations of the propulsion efficiency with *β* (*θ*_0_ = 40°, f* = 0.306,0.34,0.374).

A graph of the mean output power coefficients versus a value of *β* of *θ*_0_ = 40° for two reduced frequencies has been shown in Fig 11. It can be seen that the maximum output power coefficient increased by 15.2%, 26% and 29% for f* = 0.306,0.34,0.374 respectively. A graph of the propulsion efficiency versus a value of β of *θ*_0_ = 40° has also been given in Fig 12, from which it can be seen that the propulsion efficiencies were increased by 22.3%, 46.2% and 32.9% at values of f* = 0.306,0.34,0.374 respectively. It can be seen that under this condition, the modified pitching motion was able to effectively improve the propulsion performance of the oscillating hydrofoil.

**Fig 13:**
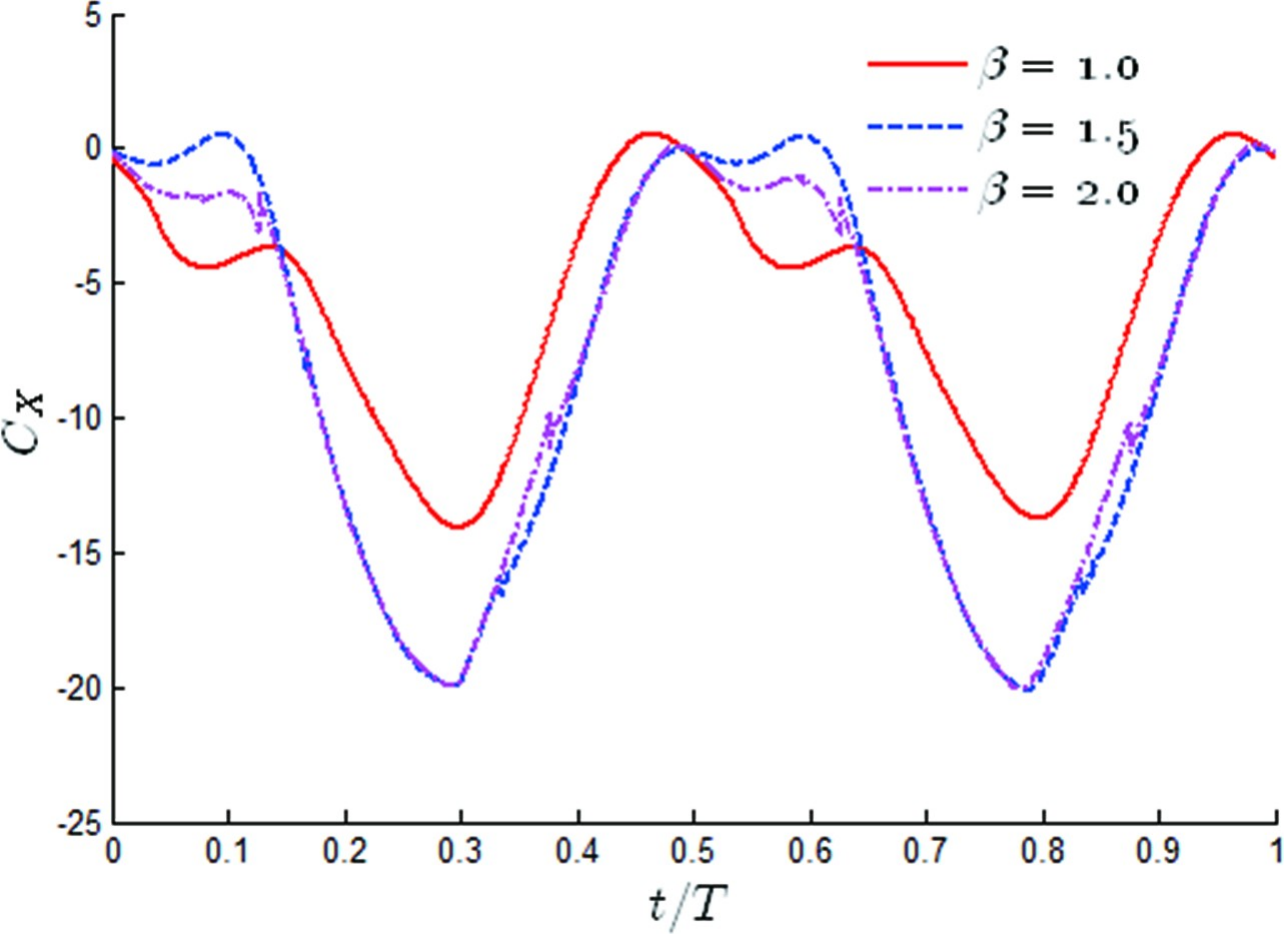
Time variations of the thrust coefficient for different values of *β* (*θ*_0_ = 40°, f* = 0.34).

In order to further understand the effects of β on the propulsion performance, the time variation over one periodic cycle of the thrust coefficients for different values of *β* has been shown in Fig 13. It was obvious that in the case of *β* = 1, the thrust coefficients had a peak twice in half a cycle, one occurring between 0–0.1t/T and one occurring at 0.3t/T. The time histories of the thrust coefficients during a cycle changed significantly when *β* = 1.5 and *β* = 2. With the increase of *β*, the propulsion performance of the oscillating hydrofoil decreased significantly between 0 and 0.1 t/T, especially at *β* = 1.5, in which the oscillating hydrofoil could not provide any propulsion. After that, the thrust coefficient rapidly increased to the maximum value. It was also noted that the highest thrust coefficient significantly increased, from 14 to 20.1 (an increase of 42.8%), and the time to reach the maximum value at *β* = 1.5 and *β* = 2 was slightly earlier than when *β* = 1.

**Fig 14:**
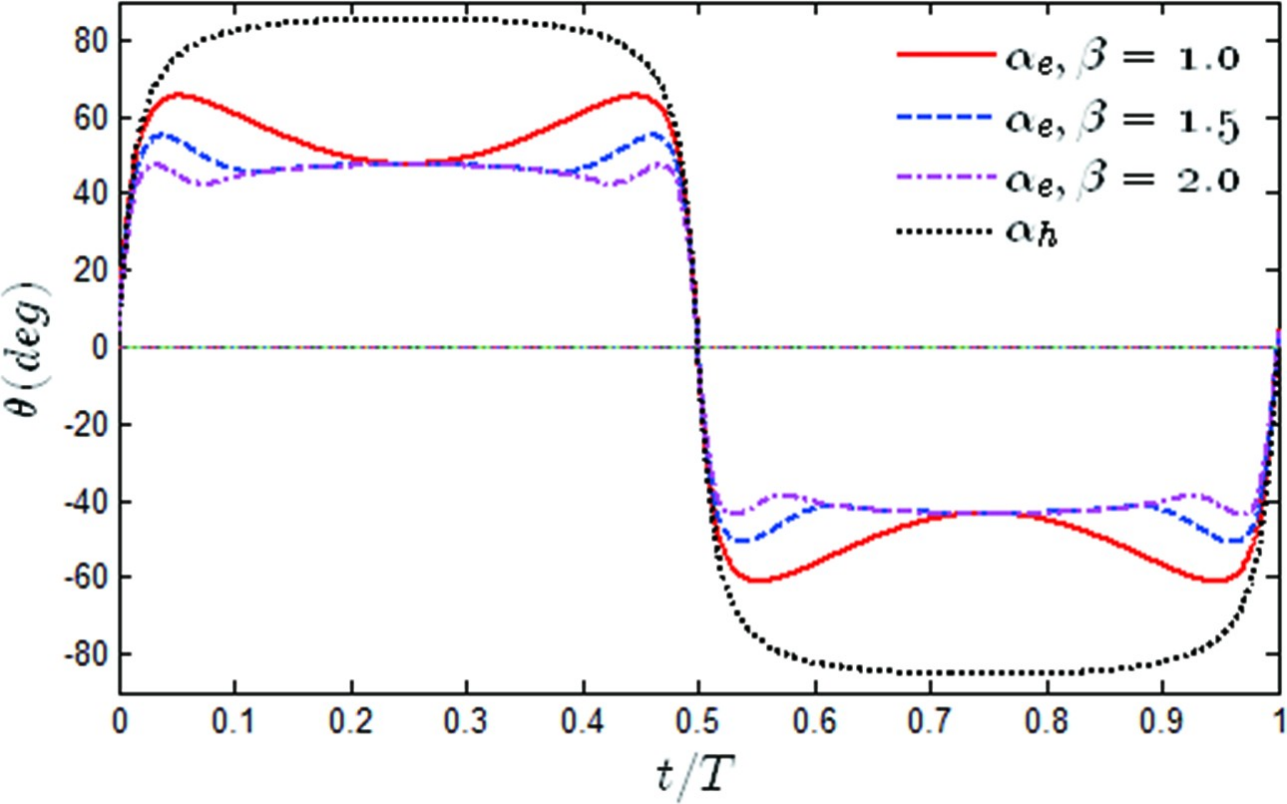
Time variations of the effective angle of attack and heaving induced angle for different values of *β* (*θ*_0_ = 40°, f* = 0.34).

The time histories of the effective angle of attack α_*e*_(*t*) and the heaving induced angle as *β* varied have been shown in Fig 14. It was found that with the increase of β, the pitching amplitude was able to achieve the extreme value more quickly than when *β* = 1, and then gradually converged to *α*_*h*_−40°. Due to the large value of α_*e*_(*t*), it could be easily observed that dynamic stall occurred during the oscillating movement. Although dynamic stall can cause boundary layer separation and has unfavorable effects on hydrodynamic performance of oscillating foil, dynamic stall typically contributes to an improvement in the propulsion performance, and the Leading Edge Vortex (LEV) has been seen to occur during the cycles of most of the efficient cases in the literature [31, 42]. Even more, they have shown that the thrust at *β* = 1 was smaller than that for *β* = 1.5 and *β* = 2 at all times. The major benefit from the persistence of α_*e*_(*t*) over a long interval was the resultant significant increase in the propulsion [26].

**Fig 15:**
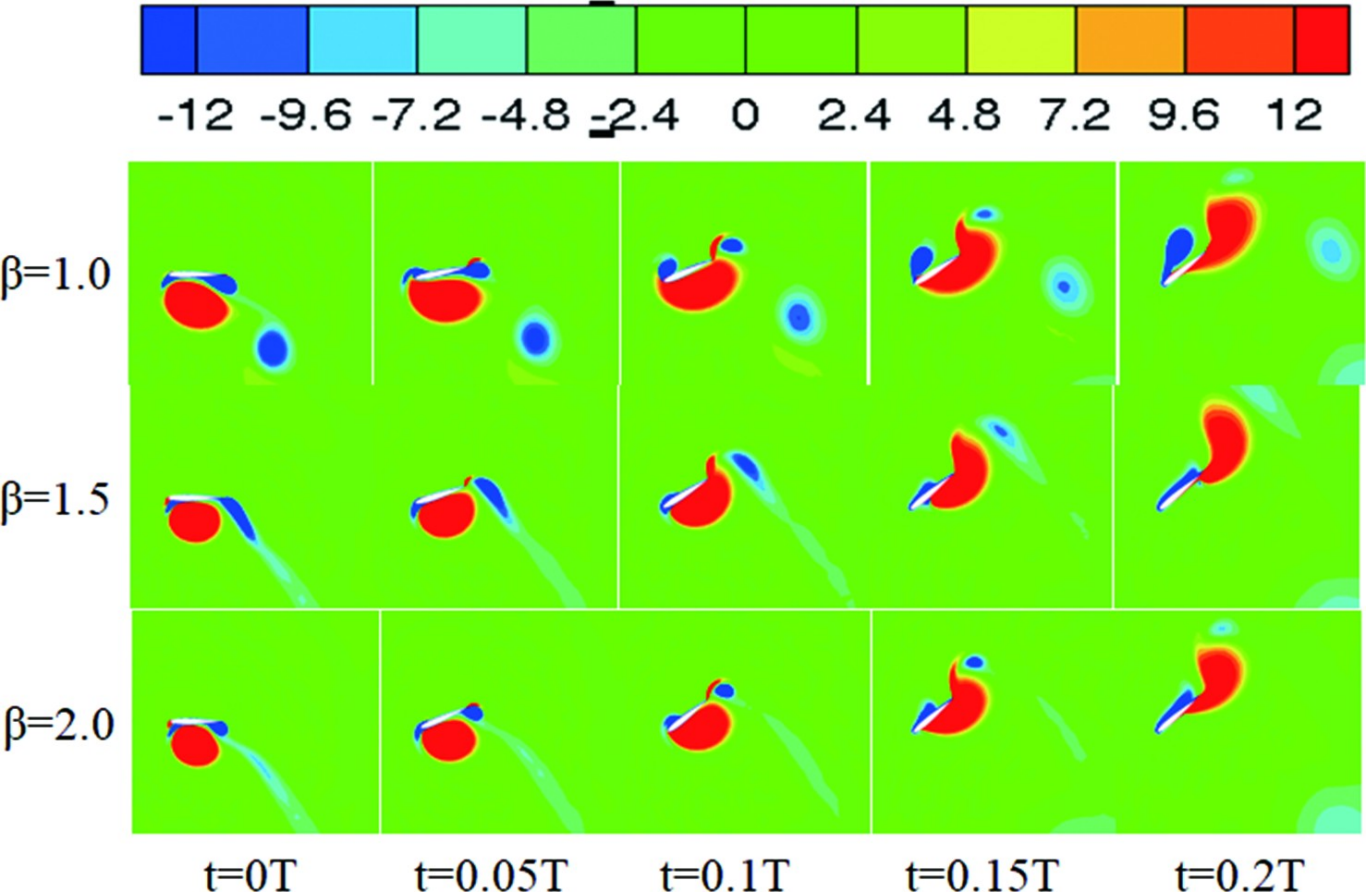
Vorticity contours for t = 0T-0.2T for different values of β (*θ*_0_ = 40°, f* = 0.34), represented by contour levels of spanwise vorticity ranging from -12(darker grey) to 12(lighter grey).

The authors wish to deeply understand the physical mechanisms of the effect of varying the values of β, in particular the effect on the time variations of the thrust coefficients. The vorticity contours that occurred between 0–0.2t/T during the period when the thrust significantly changed have been shown in Fig 15. Five instant vorticity contours have been shown in this figure. First, at t = 0.1, the LEV was generated at a value of *β* = 1, and the vorticity intensity was higher than in the other cases. This good vortex structure during this time interval resulted in the high propulsion performance of the oscillating foil. It was also noted that at this time interval the thrust coefficient for when *β* = 1.5 dropped significantly, this may be caused by the wake vortex similar to the Karmen vortex effect. Even further, it was noted that at t = 0.15T for β = 1 the LEV separated from the upper surface of the hydrofoil and the thrust increased slowly because of the vortex shedding, while when β = 1.5 and β = 2 the LEV moved downward along the hydrofoil, and it was also found that the vorticity for values of β = 2 was greater than that when β = 1.5. This revealed that the thrust coefficient rapidly increased at this time interval as shown in Fig 13. The phenomenon where the LEV attaches strongly to the surface of the hydrofoil, improving the propulsion performance, is called stall delay [26]. It was also found that the vorticity of the LEV increased with the increase of *β*.

According to the above analysis, it should be clarified that the propulsion performance of the oscillating hydrofoil was improved by modifying the profile of α_e_(t) and the topology of the vortex. However, it can be seen that the performance of the oscillating hydrofoil could be improved by tuning β for small pitching amplitude, however it could not be confirmed whether changing the value of β in the case of large pitching amplitude could improve the propulsion performance.

#### Large pitching amplitude

**Fig 16:**
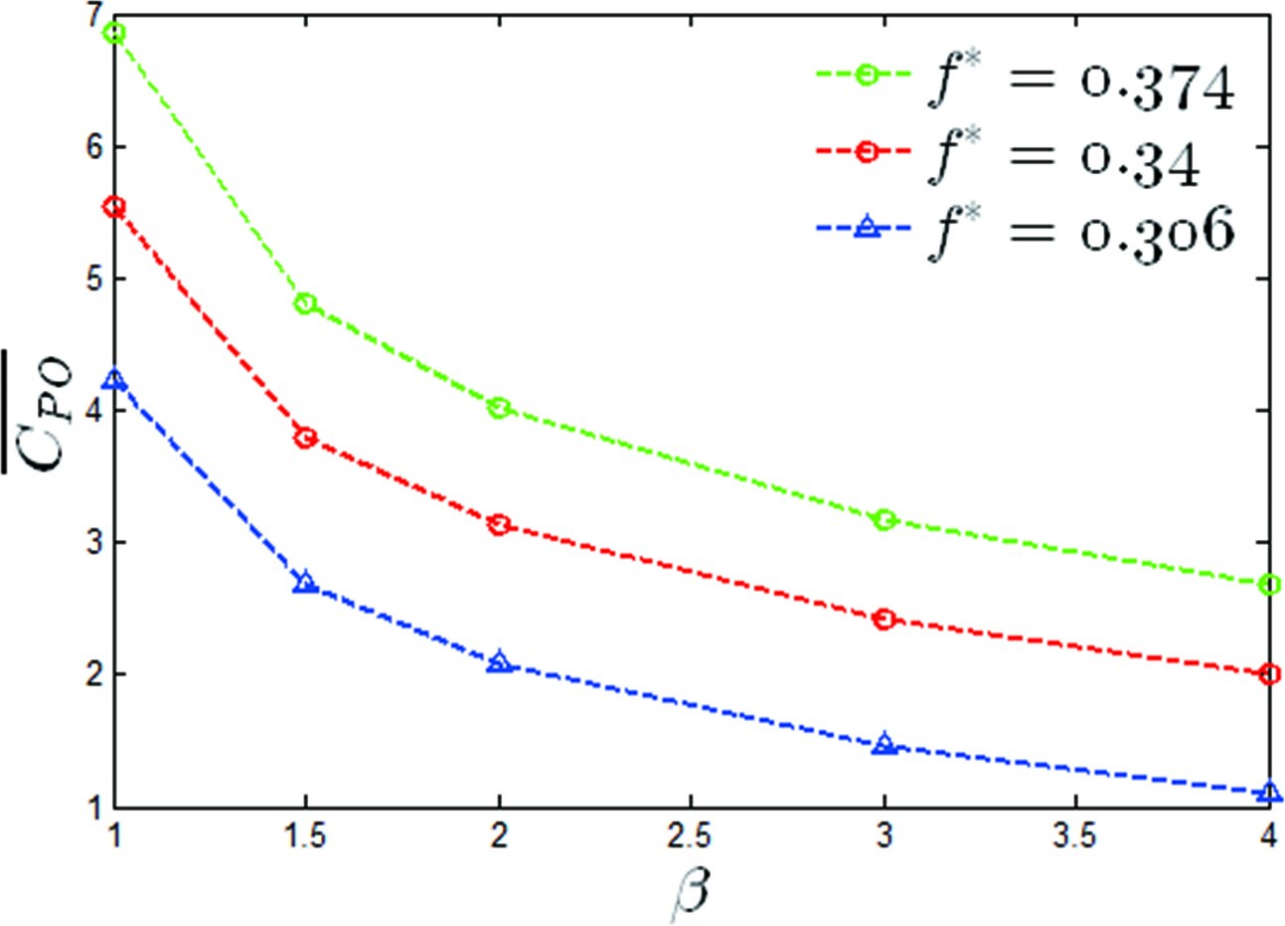
Variations of the mean output power coefficient with *β*(*θ*_0_ = 60°, f* = 0.306,0.34,0.374).

**Fig 17:**
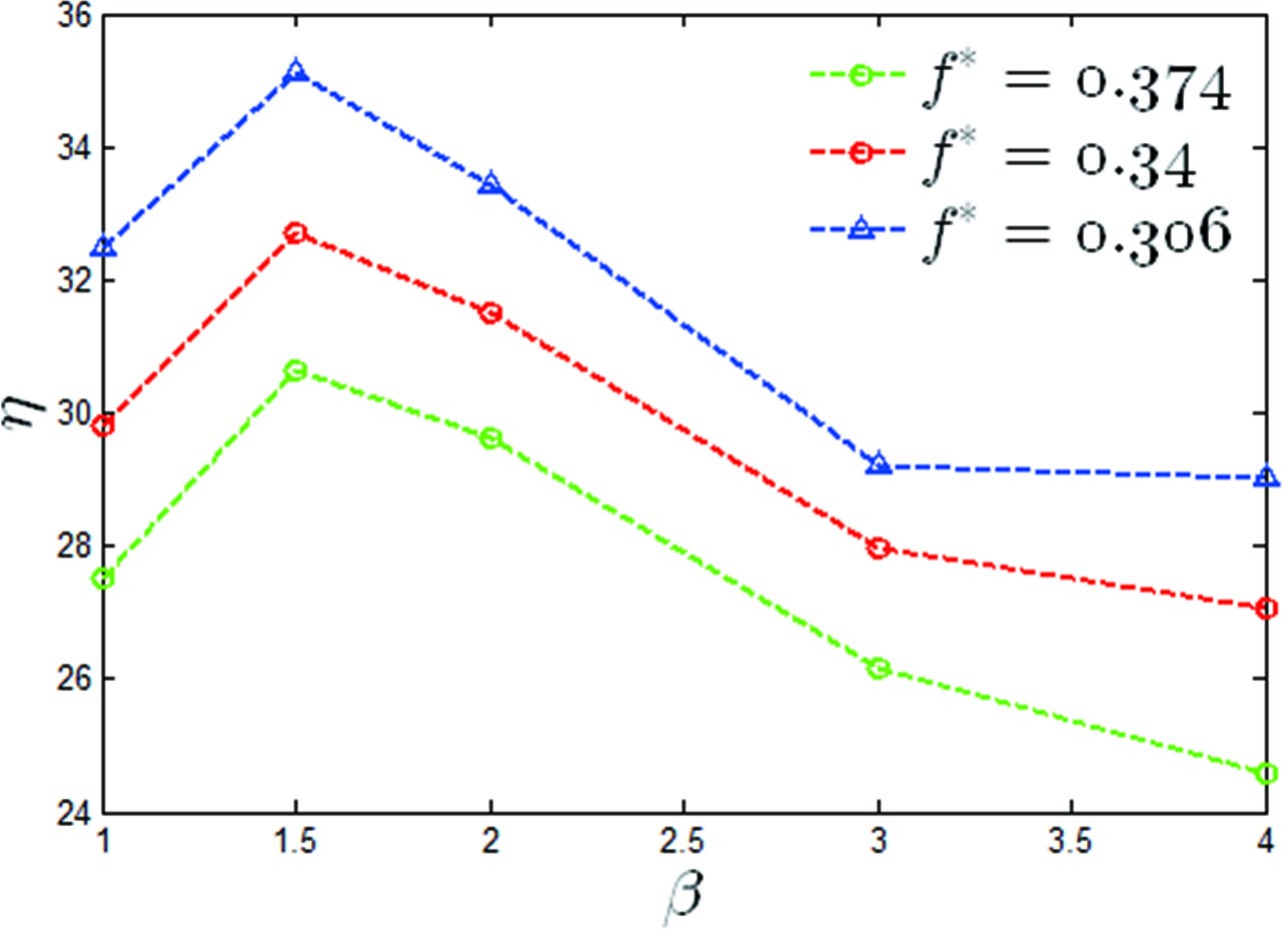
Variations of the propulsion efficiency with *β*(*θ*_0_ = 60°, f* = 0.306,0.34,0.374).

In this section, the effect of non-sinusoidal pitching motion on the propulsion performance of the oscillating hydrofoil at large pitching amplitudes has been observed. The mean output power coefficient versus β for *θ*_0_ = 60° for two heaving frequencies has been shown in Fig 16. It was noted that the maximum output power coefficients were reduced by 73.9%, 63.8% and 61% at f* = 0.306,0.34,0.374 respectively. It can be seen that for this condition, the trapezoidal-like pitching profile did not improve the propulsion performance of the oscillating hydrofoil. The propulsion efficiency for different values of *β* at *θ*_0_ = 60 have been given, and the same trend as shown in Fig 17 was found, which has proven that the propulsion efficiencies were reduced by 10.6%, 9.2% and 10.5% for f* = 0.306,0.34,0.374 respectively.

**Fig 18:**
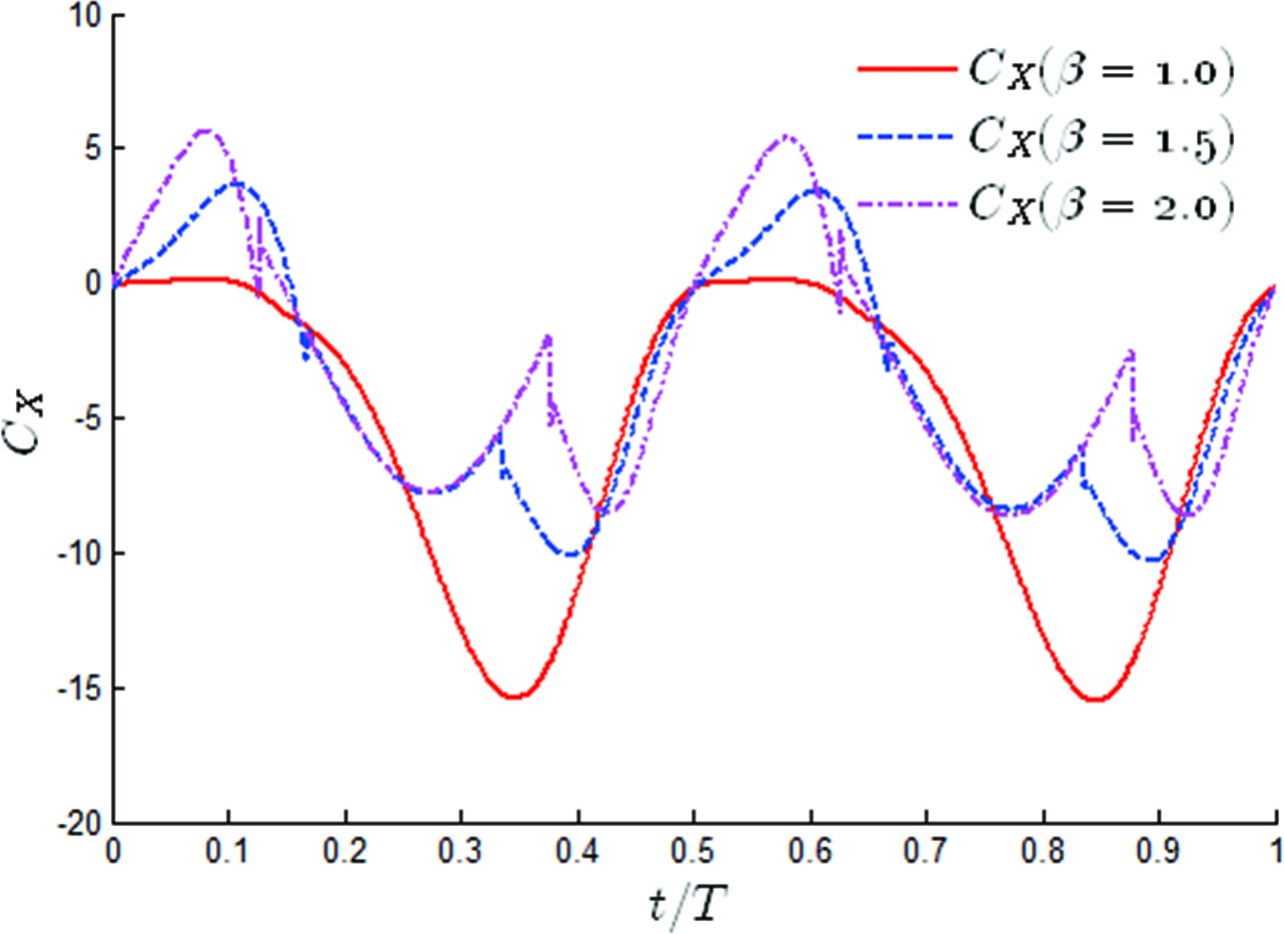
Time variations of the thrust coefficient for different values of *β* (*θ*_0_ = 60°, f* = 0.34).

Even more, they have shown that the thrusts at *β* = 1 was smaller than that for *β* = 1.5 and *β* = 2 at all times. The time history of the thrust coefficients has been given for values of β varying through *θ*_0_ = 60° in Fig 18. Through comparison with Fig 13, it was noted that there was an obvious difference in the time variations of the thrust coefficient. The time histories of the thrust coefficients both indicated that the thrust at *β* = 1.5 and *β* = 2 were smaller than that for *β* = 1 at all times. This phenomenon was the opposite of the situation at small pitching amplitudes. It was noted that in the case of *β* = 1, the thrust coefficient had one peak in half a cycle, appearing at *t* = 0.35T. The hydrodynamic performance of the oscillating hydrofoil was evidently changed by tuning the value of β. It could be observed that as the value of β increased, the time history of the thrust changed its sharp peak from a single peak to three peaks in half a cycle. Through comparison with the peak value for *β* = 1, all of the peak values were reduced. It was found that the larger the value of β, the earlier the first peak appeared and the larger the drag force. It was also noted that tuning β had little effect on the time of appearance and the peak value of the second peak, as shown in Fig 18.

**Fig 19:**
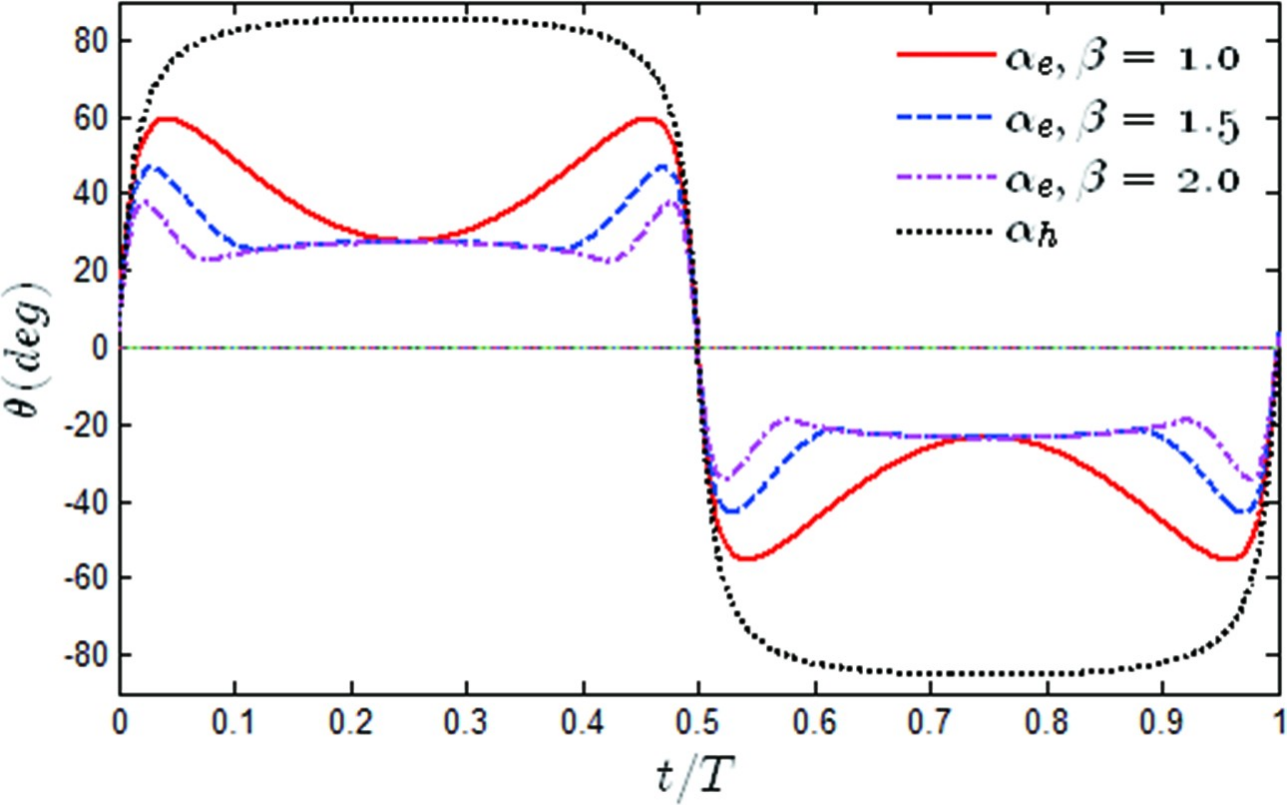
Time variations of the effective angle of attack and the heaving induced angle for different values of *β* (*θ*_0_ = 60°, f* = 0.34).

To better understand this phenomenon, the time histories of α_*e*_(*t*) as the value of β varied to *θ*_0_ = 60° have been shown in Fig 19. It was noted that the time histories of α_*e*_(*t*) had the same trend when *θ*_0_ = 60° and *θ*_0_ = 40°. Due to the increase in the pitching amplitude, α_*e*_(*t*) was significantly reduced. Although this did not cause dynamic-stall vortex shedding, a smaller α_*e*_(*t*) was not conducive to improving the propulsion performance of the flapping hydrofoil.

**Fig 20:**
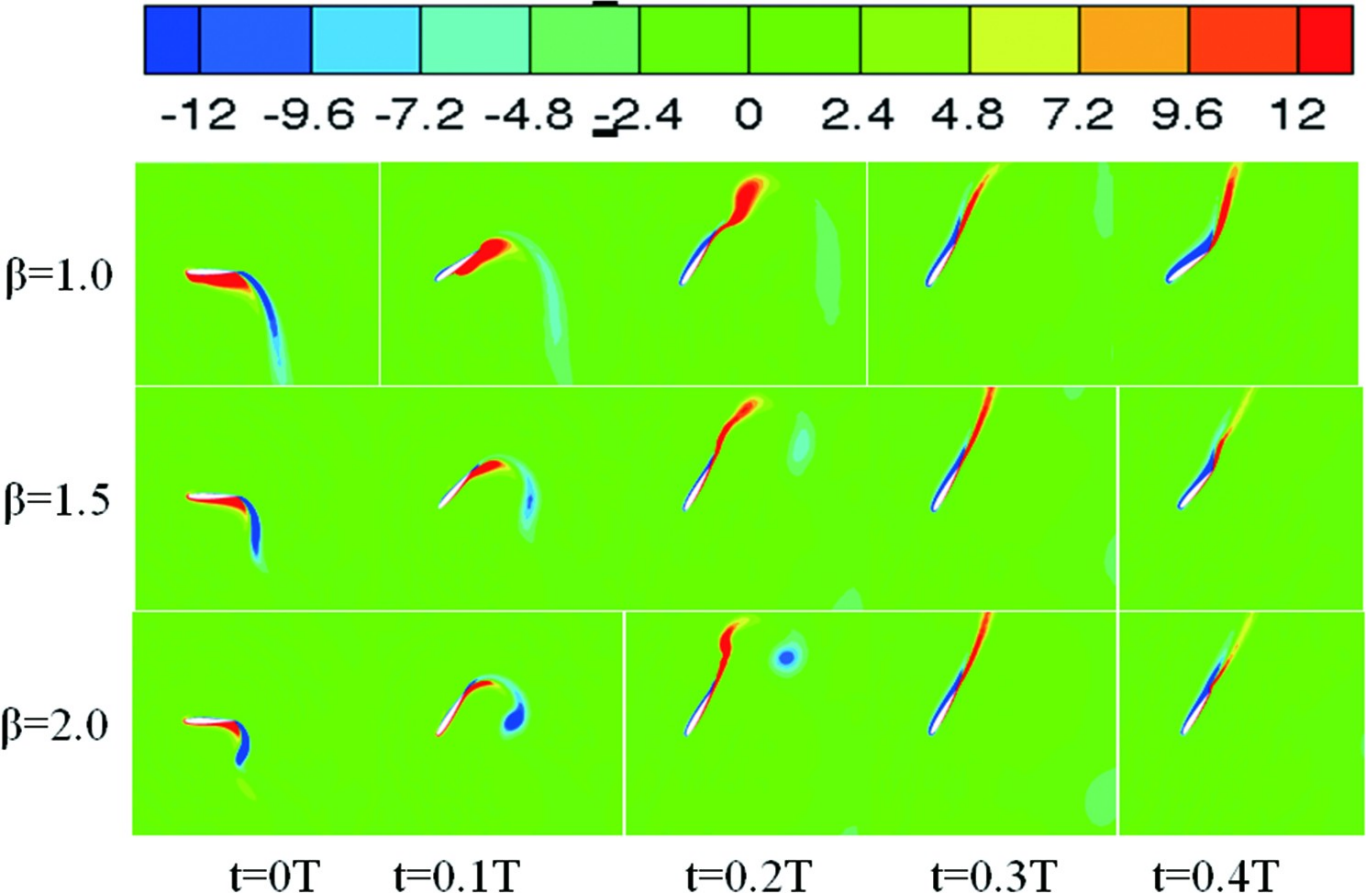
Vorticity contours during t = 0T-0.2T for different values of β (*θ*_0_ = 60°, f* = 0.34), represented by contour levels of spanwise vorticity ranging from -12(darker grey) to 12(lighter grey).

The vorticity contours significantly changed between 0–0.4t/T during the thrust and have been shown in Fig 20. Five instant vorticity contours have been shown in this figure. It can be seen that an LEV started to form around t = 0.1T for β = 1, and the LEV continued to develop and reached its largest value for values of t = 0.1–0.35T. During this time interval, no boundary layer separation occurred on the hydrofoil’s surface. It was noted that the LEV’s generation times for β = 1.5 and β = 2 were later than that for β = 1, and the vorticity intensified when β = 1.5 and β = 2 and were weaker than that for β = 1 also. It was also observed that during t = 0T-0.2T more input power was consumed in the hydrofoil’s pitching motion, delaying the generation of the LEV, and the wake vortex structure adversely affected the hydrofoil’s propulsion performance. During t = 0.3T-0.4T, it was found that the intensity of the LEV’s was enhanced, and the propulsion performance of the oscillating hydrofoil was improved.

### Full range of the pitching amplitudes

According to the discussion above, in particular, the comparison between *θ*_0_ = 40° and *θ*_0_ = 60°, it was found that for different pitching amplitudes, the effect of β produced different effects on the propulsion performance of the oscillating foil. Tuning the value of β can improve the propulsion at *θ*_0_ = 40°, and can reduce the thrust at *θ*_0_ = 60°.

**Fig 21:**
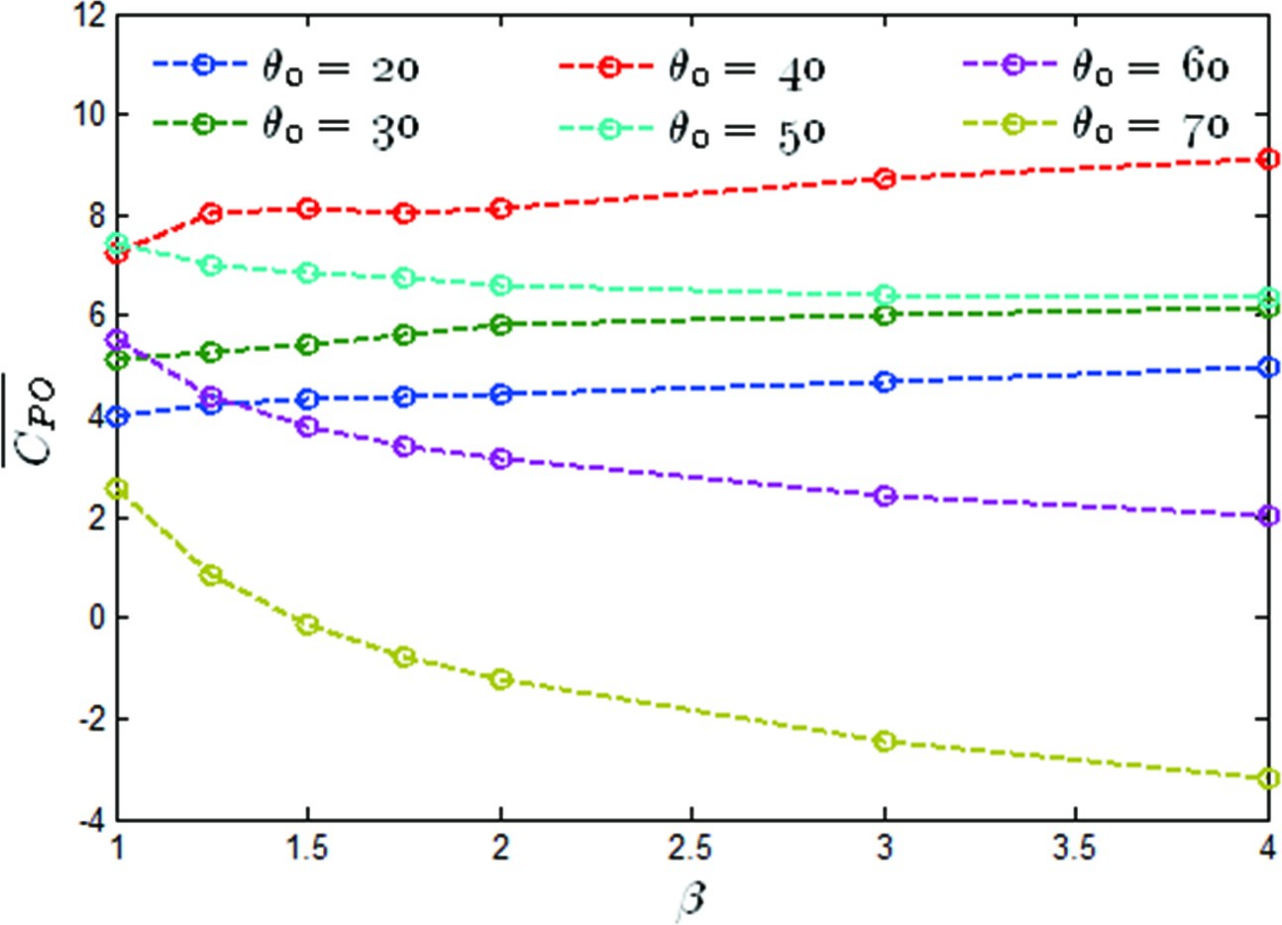
Mean output power coefficient versus β (*θ*_0_ = 20−70°, f* = 0.34).

**Fig 22:**
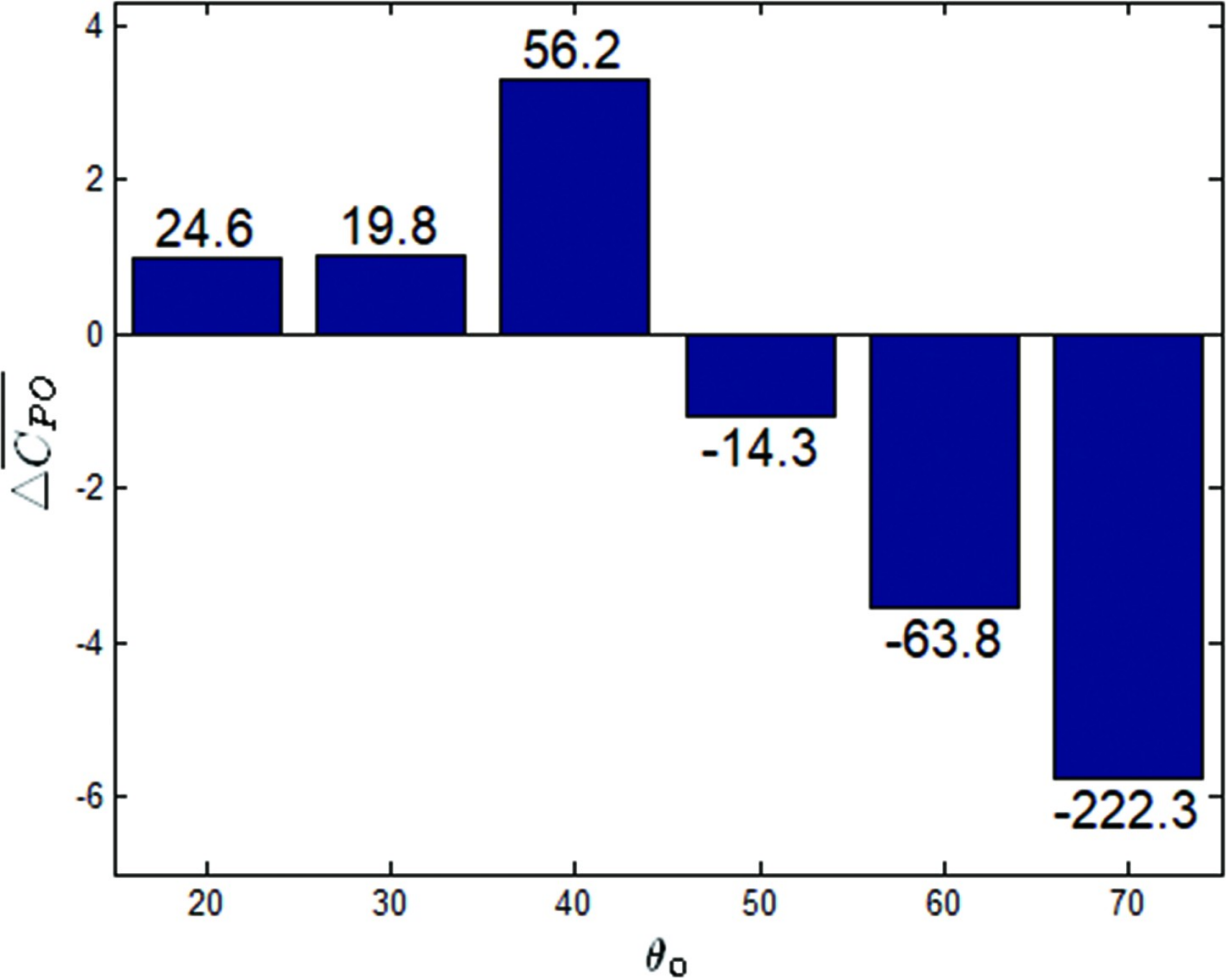
Histogram of the mean output power coefficient increments between *β* = 1 and *β* = 4 (*θ*_0_ = 20−70°, f* = 0.34).

The mean output power coefficients versus the value of β at *θ*_0_ = 20−70° have been shown in Fig 21. It was noted that for *θ*_0_ = 20°, *θ*_0_ = 30° and *θ*_0_ = 40°, the mean output power coefficients increased by 24%, 20%, and 56% respectively, and at *θ*_0_ = 50°, *θ*_0_ = 60° and *θ*_0_ = 70°, the mean output power coefficients were reduced by 14%, 64% and 222%, respectively. A histogram of the mean output power coefficient increments between *β* = 1 and *β* = 4 has been shown in Fig 22. The values above the histogram represent the percentage change in the coefficients. Based on the observations from Figs 13, 18 and 22, it can be found that the optimal pitching amplitude for f* = 0.34 was 50°. The trapezoidal pitching profile did improve the propulsion performance of the flapping hydrofoil in some case.

## Conclusions

In this study, the propulsion performance for a wave glider with an oscillating foil has been studied via two-dimensional URANS simulations. Both sinusoidal and non-sinusoidal pitching motions have been investigated in this study. First, the influence of pitching amplitude and reduced frequency on the sinusoidal pitching motion was analyzed. The results showed that as the reduced frequency increased, both the mean output power coefficient and the optimal pitching amplitude increased. Then the variations of the propulsion performance of the trapezoidal pitching motion under different pitching amplitudes have been analyzed. For small pitching amplitudes, tuning the value of β can indeed increase the propulsion efficiency, and the propulsion performance of the flapping foil gradually improved as β increased. As for the large pitching amplitude that was greater than or equal to the optimal angle of the sinusoidal pitching motion, the trapezoidal-like pitching profile could not improve the propulsion performance, and tuning the value of β significantly reduced the average thrust of the flapping hydrofoil.

The results of this study suggest that the current approach can be used to analyze the propulsion performance of an oscillating foil for a wave glider. The authors realize that the non-sinusoidal pitching profile adopted in this study is in the full-active form. However, a non-sinusoidal pitching profile in the passive form is more suitable for a wave glider and this could be considered as an opportunity for further research.

## Supporting information

S1 File The user-defined functions (UDFs) in Fluent. (PDF)

S2 File The comparison of time histories of force coefficients of the last two flapping cycles. (PDF)

S3 File Sinusoidal_pitching_motion_data. (XLSX)

S4 File Non-sinusoidal_pitching_motion. (XLSX)

S5 File Time_variations_of_CX. (ZIP)
