# Supplementary material for: Effects of non-sinusoidal pitching motion on the propulsion performance of an oscillating foil
Source: PLoS One. 2019 Jul 1;14(7):e0218832. doi: 10.1371/journal.pone.0218832 (PMC6602205; doi:10.1371/journal.pone.0218832)
Supplement: S1 File — (PDF) [file pone.0218832.s001.pdf]

```
#include "udf.h"
#define    PI      3.14
#define    F        0.55
#define    FB      1.1
#define    y0      0.5
#define    U        0.25
#define    angle    60
DEFINE_ZONE_MOTION(fmotion, omega, axis, origin, velocity, time, dtime)
{
        real T = 1/F;
        real TB = 1/FB;
        real T0 = 0;
        real T1 = TB/4;
        real T2 = T/2-TB/4;
        real T3 = T/2+TB/4;
        real T4 = T-TB/4;
        real T5 = T;
        real st0 = PI*angle/180;
        real t = time;
        real tfix = (int)(t/T);
        real tmod =t-tfix*T;
        real vy = y0*2*PI*F*cos(2*PI*F*t+PI/2);
        real dvy = y0*2*PI*F*2*PI*F*sin(2*PI*F*t+PI/2)/U;
        real s = dvy/(1+(-vy/U)*(-vy/U));
        if(tmod>=T0 && tmod<T1)
        {
                *omega = (2*FB*PI*st0)*cos(2*FB*PI*tmod );
        }
        if(tmod>=T1 && tmod<T2)
        {
                *omega = 0;
        }
        if(tmod>=T2 && tmod<T3)
        {
                *omega = (2*FB*PI*st0)*cos(2*FB*PI*(tmod -(T/2-TB/4))+PI/2);
        }
        if(tmod>=T3 && tmod<T4)
        {
                *omega = 0;
        }
        if(tmod>=T4 && tmod<T5)
        {
                *omega = (2*FB*PI*st0)*cos(2*FB*PI*(tmod -(T-TB/4))+3*PI/2);
        }
```

```
    return;
}

DEFINE_PROFILE(yinlet, thread, position)
{
    real    x[ND_ND];
    real    y;
    real    yu, K, W, PP;
    face_t    f;
    begin_f_loop(f, thread)
    {
        real    t = RP_Get_Real("flow-time");
        F_CENTROID(x, f, thread);
        F_PROFILE(f, thread, position) = -y0*2*PI*F*cos(2*PI*F*t+PI/2);
    }
    end_f_loop(f, thread)
}
```
