# Supplementary material for: Effects of non-sinusoidal pitching motion on the propulsion performance of an oscillating foil
Source: PLoS One. 2019 Jul 1;14(7):e0218832. doi: 10.1371/journal.pone.0218832 (PMC6602205; doi:10.1371/journal.pone.0218832)
Supplement: S2 File — (PDF) [file pone.0218832.s002.pdf]

The comparison of time histories of force coefficients of the last two flapping cycles (160000 cells and 1600ts/cycle) has been given. The time histories for C_X, C_Y, and C_M have been shown in Fig 23, and the numerical results of $\overline{C_{PO}}$, $\overline{C_{PI}}$ and η have been shown in Table 3.

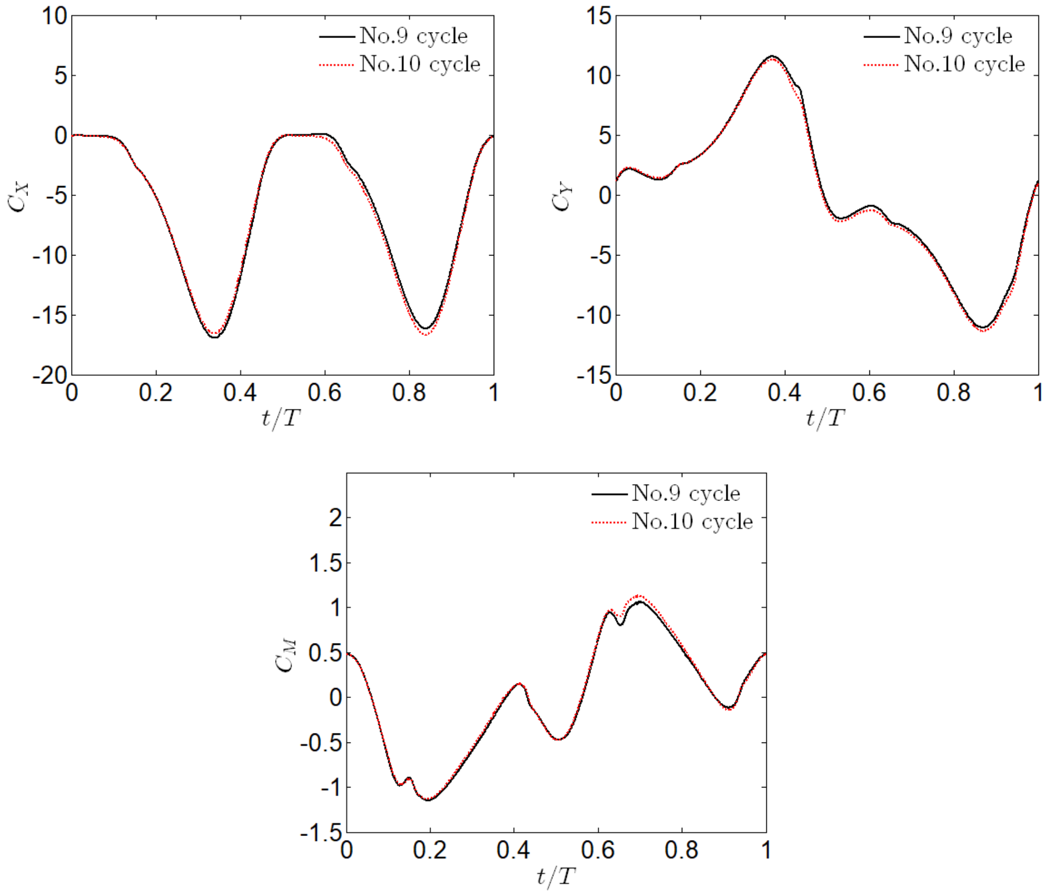

Fig 23. The time histories of force coefficients of the last two flapping cycles

Table 3. The numerical results for the different levels of cells and time steps ($\theta_0 = 57,\ f^* = 0.34$)

Cell number	Time step	Cycle number	$\overline{C_{PO}}$	$\overline{C_{PI}}$	η
160000	1600	9	6.364	22.056	28.854
160000	1600	10	6.407	22.210	28.846
